# Structure-aware retinal disentanglement reveals the genetic architecture of ocular and systemic diseases

**DOI:** 10.1371/journal.pdig.0001376

**Published:** 2026-05-15

**Authors:** Chiyu Wei, Heping Zhang, Ruibin Huang, Zelong Cai, Hongyan Wu, Chaosen Zhong, Jiong Zhang, Xiaochun Yang, Lei Sun, Xueqin Wang, Zexin Chen, Hongchao Wu, Yuqi Liu, Haizhu Tan

**Affiliations:** 1 Department of Basic Medical Sciences, Shantou University Medical College, Shantou, China; 2 North Campus of Sun Yat sen University Guangzhou Campus, Guangzhou, China; 3 Yale School of Public Health, Yale University, New Haven, Connecticut, United States of America; 4 Department of Radiology, The First Affiliated Hospital of the Medical College of Shantou University, Shantou, Guangdong, China; 5 Shenzhen Institutes of Advanced Technology, Chinese Academy of Sciences, Shenzhen, Guangdong, China; 6 Department of Gastrointestinal Surgery, The First Affiliated Hospital of Shantou University Medical College, Shantou, Guangdong, China; 7 The First People’s Hospital of Yunnan Province, The Affiliated Hospital of Kunming University of Science and Technology Kunming, Ophthalmology Department, Kunming, China; 8 Department of Ophthalmology, The Fourth Affiliated Hospital of Harbin Medical University, Haerbin, China; 9 School of Management, University of Science and Technology of China, Anhui, China; 10 Guangdong Medical University, Zhanjiang, Guangdong, China; 11 Ningbo University, Ningbo, Zhejiang, China; 12 Shanghai Customs College, Shanghai, China; QIMR Berghofer: QIMR Berghofer Medical Research Institute, AUSTRALIA

## Abstract

Deep learning effectively extracts retinal phenotypes but often functions as an entangled black box, obscuring specific genetic mechanisms and hindering clinical interpretability. To resolve this, we present the Unsupervised Ophthalmic Feature Extraction (UOFE) framework. Our approach feeds full fundus images, optic disc masks, and vessel masks into three isolated structure-aware autoencoders. Crucially, these streams share no parameters and are optimized separately to explicitly encode the macular background, optic disc, and retinal vasculature. Models were trained on 53,600 EyePACS images, utilizing a median smoothing kernel to disentangle the background, optic disc/cup, and retinal vasculature, with generalizability rigorously confirmed across 15 public datasets. Evaluated against a dimension-matched baseline trained to reconstruct the entire fundus image, UOFE demonstrated superior biological disentanglement by achieving high structural consistency between left and right eyes. In a GWAS of 75,010 UK Biobank participants, applying strict linkage disequilibrium clumping, UOFE identified 255 independent genomic loci. This represents an 8-fold increase over the monolithic baseline (31 loci) and uncovers highly novel signals compared to established global methods. Biologically, a double dissociation emerged: glaucoma mapped primarily driven by optic disc features ($P_{adj}\;<{\text{10}}^{-\text{11}}$), while AMD mapped to background features. Clinically, vascular embeddings provided powerful incremental prognostic value for diabetic retinal abnormalities (Hazard Ratio 2.38, 95% CI: 2.17–2.61, $P_{adj}\;<\text{0}.\text{0}\text{0}\text{1}$). By successfully isolating specific anatomical architectures, UOFE transforms retinal imaging into an interpretable precision window, uncovering localized genetic signals and prognostic biomarkers lost in traditional entangled representations.

## Author summary

Retinal fundus (RF) images provides a unique non-invasive window into both ocular and systemic disease. While deep learning models have enabled powerful extraction of retinal features from RF images, they typically operate as black boxes, producing entangled representations that are difficult to interpret biologically or clinically. To address this limitation, we developed the Unsupervised Ophthalmic Feature Extraction (UOFE), which separate the the retinal background, optic disc, and vasculature utilizing three structure-aware models. Each component is modeled independently to capture structure-specific features.

Using over 53,000 training images and large-scale genetic data from 75,010 UK Biobank participants, we demonstrate that this structure-aware approach improves biological interpretability and reveals distinct genetic architectures for different retinal regions. For example, genetic signals related to glaucoma were predominantly associated with optic disc features, while age-related macular degeneration was linked to background characteristics. In addition, vascular features provided meaningful prognostic information for diabetic retinal complications.

Hence, our proposed UOFE framework provides a more precise tool for discovering biological and genetic mechanism and clinically relevant risk stratification.

## Introduction

The human retina provides a unique, non-invasive imaging window into the vascular and neural status of the body, enabling large-scale investigation of complex ocular and systemic diseases. In particular, retinal phenotypes extracted from Retinal fundus (RF) images serve as critical biomarkers for major pathologies such as glaucoma, cataract, and diabetic retinal abnormalities. Given the high heritability of these traits, discovering the genetic architecture underlying retinal morphology is essential for unraveling disease etiology and developing predictive risk models [1,2]. However, most existing image-derived features (IDFs) used in genome-wide association studies (GWAS) are based on expert-defined or heuristic labels. Such rigid definitions often fail to capture the continuous, high-dimensional spectrum of retinal variations, limiting phenotypic resolution and overlooking subtle indicators of pathophysiology [3].

Previous large-scale GWAS have demonstrated the potential of RF imaging for genetic discovery. For instance, genetic analyses of vessel tortuosity [4] and optic disc morphology [5] have identified numerous loci linked to vascular and neural phenotypes. Despite these advances, current methods face three major challenges. First, the structural heterogeneity of the retina is often oversimplified [6]. Second, existing IDFs predominantly describe coarse-grained anatomical features, and failing to capture micro-structural variations relevant for disease [7]. Third, reliance on manual annotation imposes a bottleneck on scalability and introduces inter-observer variability. To address these limitations, unsupervised deep learning offers a promising alternative by enabling automated feature extraction directly from raw images [8].

Deep learning approaches offer a promising alternative by automating feature extraction directly from raw images. While supervised methods, such as TransferGWAS [9], have demonstrated success, they depend heavily on expensive expert annotations and are inherently limited to known disease features. Conversely, self-supervised frameworks like iGWAS [10]) circumvent the need for labels but often yield globally entangled high-dimensional embeddings. These representations lack anatomical specificity, mixing signals from distinct structures (e.g., optic disc and background) into a ‘black box’ latent space [11,12], which hinders the biological interpretation of genetic associations. Unsupervised generative models, particularly Variational Autoencoders (VAEs) [13], provide a promising alternative by learning compact, probabilistic representations. However, standard VAE approaches typically model the retinal fundus image as a single, indivisible entity, a strategy we term the Monolithic approach. By compressing the entire image into a shared latent space without structural differentiation, this monolithic strategy suffers from a critical limitation: the global reconstruction is often dominated by low-frequency background signals. Consequently, high-frequency details essentials for clinical diagnosis, such as peripheral micro-vasculature or the precise cup-to-disc boundary, frequently attenuated or lost in the reconstruction process [14]. These coarse entangled representations lack the sensitivity required to detect the subtle, localized phenotypic variations driven by genetic variants.

To address these challenges, we developed the Unsupervised Ophthalmic Feature Extraction (UOFE) framework, anchored by a novel Structure-Aware VAE (SA-VAE) architecture. Diverging from monolithic approaches, SA-VAE adopts a substructure-disentangled strategy. By explicitly segmenting the retina into distinct anatomical domains (vasculature, optic disc/cup, and background), the model learns dedicated, biologically meaningful representations focusing on each structure. This design enables the high-fidelity capture of fine-grained phenotypes (e.g., vessel tortuosity) typically obscured in global embeddings. We rigorously validated the framework through a multi-modal pipeline: assessing feature disentanglement via quantitative metrics (DCI); uncovering the underlying genetic architecture through multi-trait genome-wide association studies (GWAS) and Linkage Disequilibrium Score Regression (LDSC); and evaluating clinical utility by modeling the risk of major ocular and systemic diseases (glaucoma, cataract, and diabetic retinal abnormalities) using a Lasso-penalized Cox proportional ha5zards model. Collectively, this study demonstrates that UOFE achieves superior reconstruction fidelity while revealing novel genetic associations and robust predictive biomarkers, establishing a scalable, label-free paradigm for precision retinal phenotyping.

## Results

### Study population and data characteristics

**Model development cohort.** To ensure robust feature learning, the primary training corpus was constructed from the EyePACS dataset, a large-scale (N > 88,000) and highly heterogeneous cohort that provides the essential structural variance required to train stable unsupervised representations [15]. Following rigorous quality control (QC), a final set of 53,600 high-quality images was retained from the EyePACS dataset.

We applied the trained models to the UK Biobank (UKB) [16] for large-scale feature extraction (N = 130,329). To rigorously validate reconstruction fidelity and cross-cohort generalization, we compiled a diverse pool of ~170,000 images from 15 independent public datasets. Due to the computationally intensive nature of specific quantitative metrics (e.g., LPIPS and disentanglement scoring), the out-of-sample evaluation was performed on a representative test set of 1,000 images randomly sampled from this full compound dataset. For the genomic association analyses, the UKB population was specifically refined to 75,010 participants possessing paired high-quality retinal imaging and genotype data. Following strict genotype imputation and filtering, 5,261,211 genetic variants were retained for GWAS.

Demographic Heterogeneity. As detailed in Table 1, statistically significant disparities were observed between native and non-native English speakers across all baseline characteristics (P<0.001; significance threshold, α = 0.05). Notably, the native English group was characterized by a slightly advanced age (56.01 vs. 53.68 years) and a higher prevalence of smoking. These demographic differences underscore the necessity of rigorous covariate adjustment (including age, sex, and BMI) in subsequent genetic and clinical analyses to mitigate potential confounding effects.

**Table 1:** Baseline Characteristics of the Study Population Stratified by Ethnic Background.

Characteristic	White Participants (n = 34,728)	Non-White Participants (n = 3,374)	*P*-value
Age (years)	56.01 ± 8.09	53.68 ± 8.49	<0.001
BMI (kg/m²)	27.19 ± 4.69	26.84 ± 4.71	<0.001
Sex			<0.001
Female	18,958 (54.6%)	1,980 (58.7%)	
Male	15,770 (45.4%)	1,394 (41.3%)	
Smoking status, n (%)			<0.001
Never smoker	92 (0.3%)	15 (0.4%)	
Current smoker	19,553 (56.3%)	1,630 (48.3%)	
Former smoker	11,961 (34.4%)	1,296 (38.4%)	
Unknown	3,122 (9.0%)	433 (12.8%)	
Alcohol consumption, n (%)			<0.001
Regular drinker	17 (0.0%)	7 (0.2%)	
Occasional drinker	1,012 (2.9%)	133 (3.9%)	
Never drinker	1,097 (3.2%)	139 (4.1%)	
Unknown	32,602 (93.9%)	3,095 (91.7%)	

Notes: Data are presented as mean (SD) or N (%). P-values compare characteristics between [Group A] and [Group B] using t-tests for continuous variables and chi-square tests for categorical variables.

### Structure-aware disentanglement enhances reconstruction fidelity

**Fig 1:**
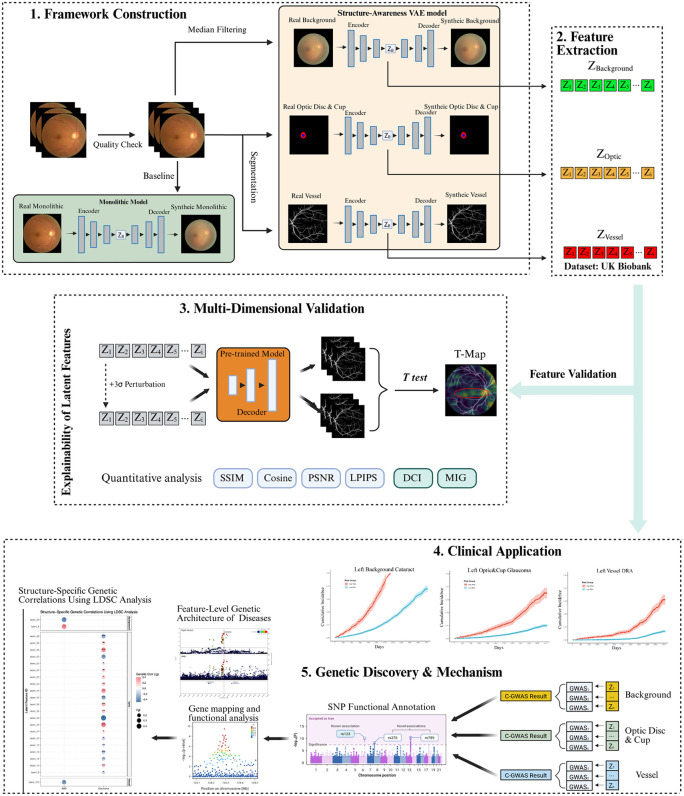
Overall study design and the Unsupervised Ophthalmic Feature Extraction (UOFE) framework. Notes: (1) Framework Construction: Retinal images undergo rigorous quality checks and are processed via two pathways: a standard Monolithic model (encoding the whole image) and the proposed Structure-Aware VAE (SA-VAE) model, which explicitly encodes pre-segmented anatomical domains (background via median filtering, optic disc/cup, and vessels). (2) Feature Extraction: Low-dimensional, structure-specific latent representations (Z_Background_, Z_Optic_, Z_Vessel_) are extracted from a large-scale population cohort (UK Biobank). (3) Multi-Dimensional Validation: The learned latent features undergo strict evaluation. This includes quantitative performance metrics (e.g., SSIM, LPIPS, DCI) and qualitative perturbation experiments to verify biological interpretability and feature disentanglement. (4) Clinical Application: Prognostic validation of the extracted features. Kaplan-Meier survival curves illustrate robust disease risk stratification. (5) Genetic Discovery & Mechanism: Integration of latent features into multi-trait C-GWAS to uncover novel genetic loci, followed by post-GWAS analyses (e.g., LDSC genetic correlation, functional annotation) to reveal the feature-level genetic architecture of the human retina.

To rigorously validate the substructure-disentangled strategy, we benchmarked the SA-VAE framework against Monolithic VAE baseline on the identical external validation set of 1,000 images. Quantitative evaluation (see Table B in S3 File and Fig K–Fig P in S2 File) revealed that the SA-VAE models achieved superior reconstruction fidelity compared to the Monolithic baseline. To ensure statistical robustness, all metrics were averaged across 10 independent resampling iterations; the resulting exceptionally low standard deviations confirm the high stability and reproducibility of the learned representations (Fig 1).

**Fig 2:**
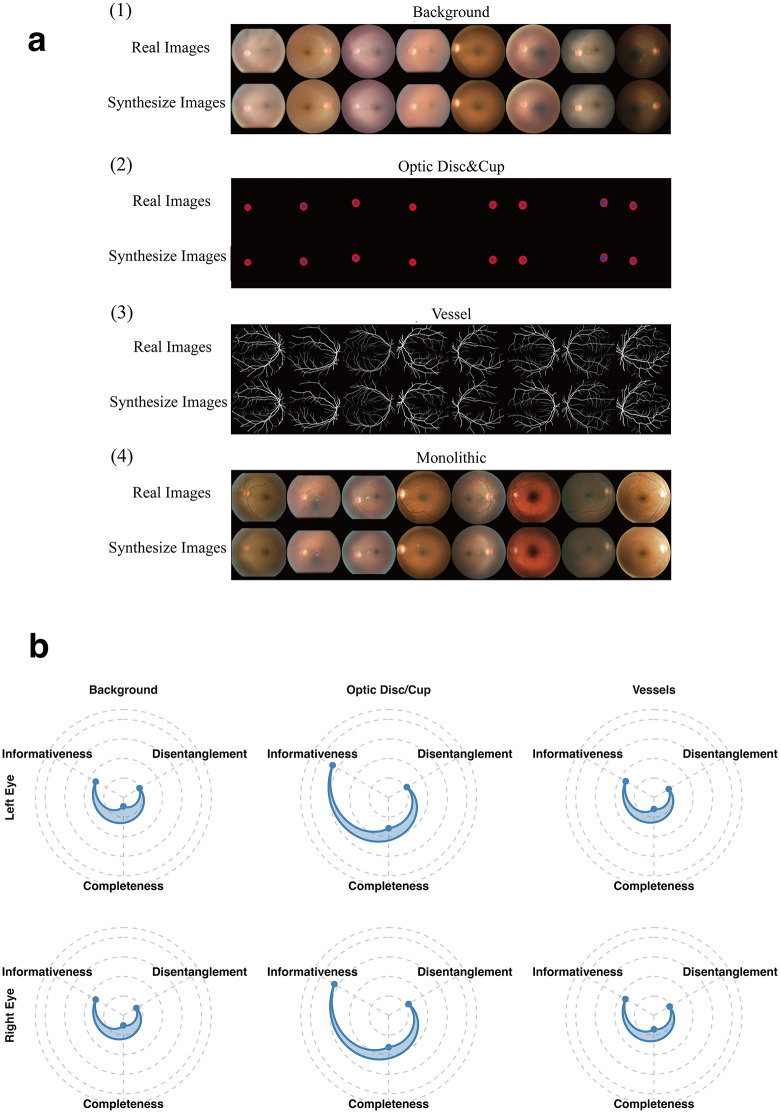
Qualitative and Quantitative Evaluation of Reconstruction Fidelity and Feature Disentanglement. Notes: **(a)** Visual Reconstruction Comparison. Representative input images (top row) and their reconstructions (bottom row) generated by the structure-specific UOFE models versus the Monolithic Baseline. Note that the Monolithic model (Panel 4) produces blurred vascular details, whereas UOFE preserves fine-grained topologies. **(b)** Disentanglement (DCI) Radar Charts. Radar plots displaying Disentanglement (D), Completeness (C), and Informativeness (I) scores for each substructure across Left (blue) and Right (red) eyes. The high overlap between eyes confirms model robustness. The high Informativeness of Optic features (>0.8) contrasts with the distributed encoding of Background textures, validating the structure-aware separation strategy.

Specifically, the Optic Disc and Background sub-models demonstrated near-perfect perceptual quality, with LPIPS scores consistently below 0.04 and SSIM exceeding 0.96 across both eyes. This represents a dramatic improvement over the Monolithic baseline, which struggled with global optimization trade-offs (e.g., Monolithic LPIPS ≈0.21). While the dedicated Retinal Vessel model yielded lower pixel-based metrics (SSIM ≈ 0.30, PSNR ≈ 13–15 dB). However, this apparent performance gap is an expected consequence of how these standard metrics evaluate very thin, branching structures. Unlike the broad and dense background areas, the capillary network occupies only a tiny fraction of the image pixels. Therefore, traditional pixel-matching metrics (like MSE or SSIM) will heavily penalize the model for even microscopic (sub-pixel) spatial shifts, even if the overall biological shape, branching, and tortuosity of the vessels are perfectly preserved. Crucially, the perceptual LPIPS metric remained highly competitive (≈0.49–0.54), confirming that the SA-VAE framework successfully captures the fine-grained vascular complexity and tortuosity that are typically attenuated or blurred within the global information bottleneck of Monolithic representations (Fig 2a).

Furthermore, DCI analysis (Fig 2b and Table C in Supplementary Data) quantitatively confirmed the success of feature disentanglement. The radar plot reveals distinct and functionally appropriate metric profiles across the three anatomical sub-models. Specifically, the Optic Disc/Cup features achieved remarkably high Informativeness scores (>0.80). This indicates that the latent dimensions within this sub-model efficiently capture a dense, highly predictive mapping to known geometric clinical biomarkers (e.g., cup-to-disc ratio or neuro-retinal rim width). Conversely, the Background and Vessel features exhibited highly robust Disentanglement scores that were almost identical between independent left and right eye inputs (e.g., Left Vessel D-score: 0.417 vs. Right: 0.421).

This structural independence and high inter-ocular consistency confirm that the SA-VAE framework successfully forces the respective models to isolate sparse vascular topologies and photometric textures into orthogonal latent dimensions. It is typically unattainable in standard entangled, monolithic architectures. While differing reconstruction objectives between our model and the monolithic baseline preclude attributing the superior reconstruction fidelity solely to anatomical compartmentation, these findings strongly demonstrate that our proposed SA-VAE model, combining explicit structural separation with generative VAE objectives, effectively preserves true biological specificity.

### Qualitative assessment of feature interpretability

**Fig 3:**
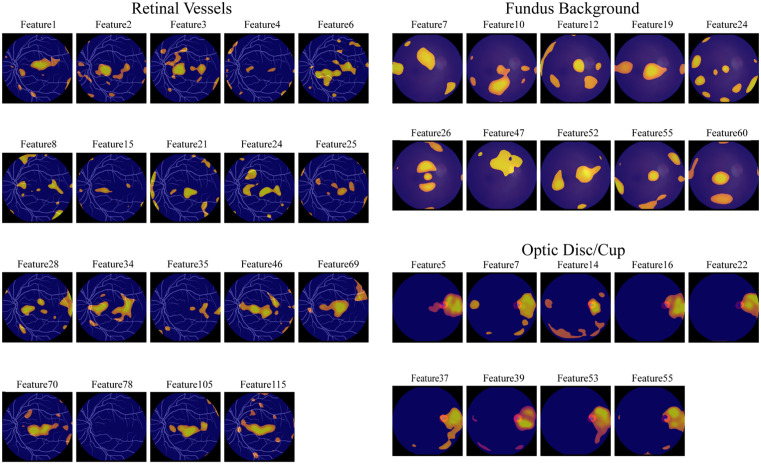
Qualitative Interpretability of Latent Features via Spatial Perturbation. Notes: Fig 3 illustrating the specific retinal regions modulated by individual latent dimensions across the three SA-VAE sub-models. Yellow/red regions indicate areas with the highest pixel-level variance when a single corresponding latent feature is systematically perturbed. **(a)** Retinal Vessels: Vessel-specific features demonstrating region-specific control over vascular topology. Distinct features independently encode localized microvascular regions, such as the arcuate pathways (e.g., Feature 1, 46) or the peripheral arcades (e.g., Feature 4, 69). **(b)** Fundus Background: Background-specific features capturing photometric characteristics. Perturbations map to spatially distributed, non-vascular regions, reflecting independent control over global illumination gradients, macular pigmentation, and background tessellation patterns. **(c)** Optic Disc/Cup: Optic disc-specific features displaying highly localized geometric specificity. The perturbation responses are strictly confined to the structural boundaries of the optic nerve head, indicating precise encoding of local morphology (e.g., cup-to-disc ratio and neuro-retinal rim) without entangling with surrounding vascular or background components.

To complement the quantitative evaluation, we conducted latent space perturbation experiments to examine the biological semantics of individual IDFs (Fig 3; Fig H–Fig J in S2 File). We assessed whether the SA-VAE models decompose retinal information into coherent and functionally meaningful components.

Retinal Vessels (Topological Specificity). Perturbations of vessel-related latent dimensions revealed a clear, region-specific control over vascular topology. For example, Features 1 and 46 predominantly affected the vasculature along the arcuate pathways and in the peripapillary region, increasing or decreasing vessel crowding around the optic disc without inducing widespread changes elsewhere in the fundus. Other dimensions, such as Features 4 and 69, showed stronger effects along the more peripheral vascular arcades; inspection of the corresponding reconstructions indicated coordinated alterations in vessel tortuosity and caliber in these distal regions.

Optic Disc and Cup (Geometric Specificity). Perturbations in the SA-VAE latent space primarily modulated geometric parameters. Changes in these IDFs altered the cup-to-disc ratio, neuro-retinal rim width, and disc ovality, while the surrounding vasculature and background appearance remained largely stable. The corresponding saliency maps showed responses confined to the disc and cup region, indicating that this module encodes local geometric morphology rather than global image style.

Fundus Background (Photometric Specificity). Background‑related IDFs primarily captured photometric and textural characteristics. Perturbations produced smooth shifts in global illumination gradients, macular pigmentation density, and background tessellation patterns. The optic disc appeared as a high‑intensity area in several background saliency maps, reflecting its high reflectance in the median‑filtered input. However, these perturbations mainly modulated local brightness and reflectivity around the disc and did not systematically reshape disc or cup boundaries. This observation supports a functional separation between modules: the VAE_Background_ encodes the optic disc as a high‑albedo region within the overall luminance field, whereas the VAE_disc-cup_ represents it as a structural landmark characterized by clinically relevant geometric parameters.

### Structure-aware disentanglement amplifies genetic discovery

To characterize the genetic architecture of the learned phenotypes, we performed multi-trait C-GWAS analyses. We rigorously benchmarked the discovery power of our structure-aware UOFE framework against two distinct baselines: a state-of-the-art Vision Transformer (ViT) and an ablation Monolithic VAE baseline (trained on whole, unsegmented images). All comparisons were evaluated on the identical set of preprocessed images.

**Fig 4:**
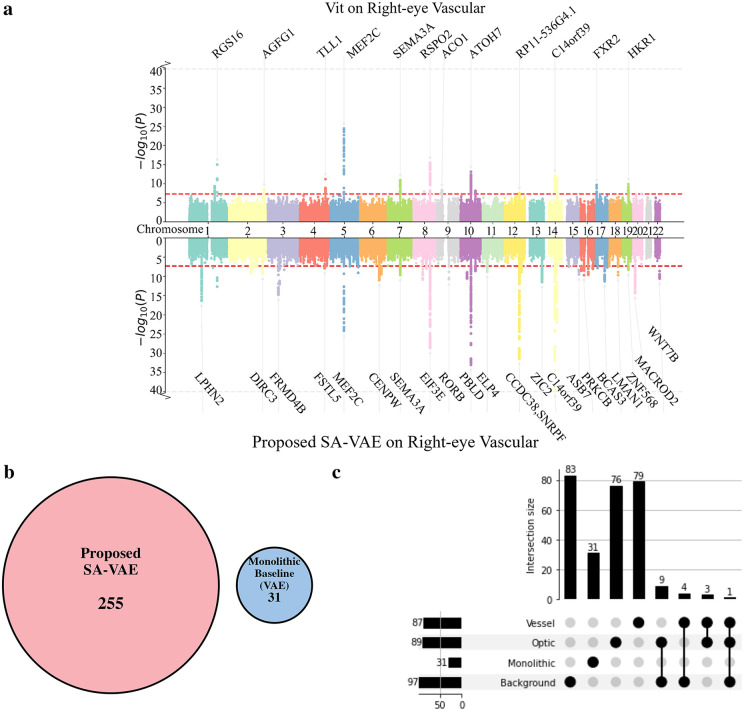
Genomic Discovery: Benchmarking the Structure-Aware (SA-VAE) Framework. Notes: **(a)** Comparison with Vit. Miami plot depicting genome-wide association results for matched 128-dimensional latent feature spaces extracted from right-eye vascular images. The upper panel displays the association landscape derived from the monolithic ViT baseline, while the lower inverted panel shows the markedly enriched signals recovered by our proposed SA-VAE framework using an identical number of variables. Red dashed lines indicate the threshold for genome-wide significance (P < 5 × 10 ^−8^). **(b)** Validation of Structural Disentanglement. To represent the full SA-VAE framework, latent vectors from the three independent streams were concatenated into a single, unified feature vector. This was compared against a monolithic VAE baseline. Numbers indicate the independent genomic loci (after strict LD clumping) uniquely captured by each model. **(c)** Upset plot detailing the specific intersection of these independent loci across the three SA-VAE sub-models and the Monolithic VAE baseline. The matrix dots indicate intersecting sets, and the top bars display the count of independent loci per intersection. The red dashed line indicates the threshold for genome-wide significance (P < 5 × 10^-8^). The grey dashed line serves solely as a visual aid to indicate the position of the y-axis break.

First, we benchmarked the proposed framework against a standard ViT architecture. Crucially, to ensure a fair comparison unaffected by representation size, both models were configured to extract identically sized 128-dimensional latent spaces from the exact same right-eye vascular images (detailed ViT architecture and training setups are provided in the S1 File). As illustrated in the Miami plot (Fig 4a), UOFE demonstrated a substantial advantage under these matched conditions. It recovered a significantly richer association landscape with sharper peaks and broader genomic coverage compared to the ViT baseline, establishing its superior overall genetic discovery power.

To validate that this performance gain stems specifically from our anatomical decomposition strategy, we first benchmarked the UOFE (SA-VAE) framework against a monolithic VAE baseline. To strictly control for the confounding effects of LD, all comparisons were evaluated at the level of independent genomic loci(r^2^ < 0.1, 250 kb window). Under these rigorous criteria, our framework identified a total of 255 independent loci, representing an approximately 8-fold increase compared to the monolithic baseline, which detected only 31 loci in the identical cohort (Fig 4b). Notably, these two sets of loci were completely disjoint, sharing zero overlap. This profound divergence is driven by the structural specificity of our approach: as demonstrated by the UpSet plot (Fig 4c), the vast majority (>93%) of the 255 loci discovered by SA-VAE mapped primarily driven by a single anatomical domain (83 to the Background, 76 to the Optic Disc, and 79 to the Retinal Vessels), with minimal pleiotropic overlap across structures.

To further contextualize our findings against current state-of-the-art approaches, we formally benchmarked UOFE against the established iGWAS method. While iGWAS identified 113 independent loci in the same dataset, the overlap with our 255 loci was remarkably minimal, with only 4 shared signals (Fig A in S1 File).

Together, these quantitative results demonstrate that SA-VAE model successfully isolates high-frequency, structure-specific genetic signals that are fundamentally obscured when entangling the entire image into a standard monolithic global analysis. (Detailed SNP-based heritability estimates for all single latent variables and intercepts are provided in Fig Q–Fig T in S2 File).

### Structure-specific genetic architectures and biological validity

**Fig 5:**
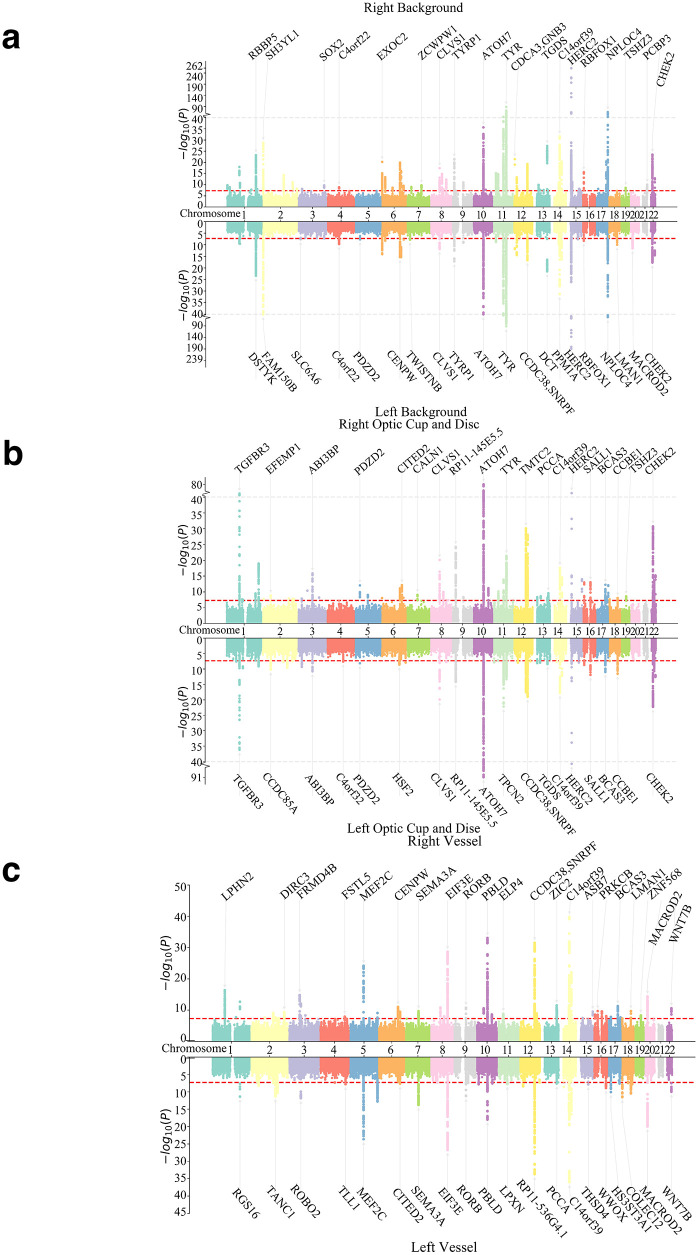
Genome-wide associations for retinal substructures. Notes: The red dashed line indicates the threshold for genome-wide significance (P < 5 × 10^−8^). The grey dashed line serves solely as a visual aid to indicate the position of the y-axis break.

Stratifying GWAS results by anatomical domain revealed distinct, biologically coherent genetic profiles, confirming that the learned phenotypes capture non-redundant biological information (Fig 5, Table D–Table L in S3 File and Fig U in S2 File). First, the Optic Disc/Cup features were strongly enriched for loci involved in neuroretina development, successfully replicating established associations at *TGFBR3*, *SIX6*, and *ATOH7*. Admittedly, supervised models explicitly trained to quantify specific clinical metrics, such as the vertical cup-to-disc ratio (VCDR) [17], yield higher statistical power for those isolated traits, identifying over 140 loci compared to the 53 loci discovered by our unsupervised optic disc module. However, traditional summary statistics like VCDR inherently conflate various complex morphological factors into a single ratio. In contrast, our latent feature analysis allows for a substantially more granular dissection of the optic disc’s genetic architecture. For instance, while *ATOH7* is classically associated with overall optic disc size [18], our model isolated its specific impact on geometric rim parameters, entirely independent of cup depth.

In parallel, distinct genetic signals emerged from the Retinal Vessel and Background modules (Fig U in S2 File). Retinal Vessel features mapped specifically to angiogenesis and endothelial pathways, characterized by prominent peaks at MEF2C, C14orf39, and FGF5. Conversely, Background features were linked to pigmentation and metabolic genes, such as TYR and HERC2. As illustrated in Fig 5, these pigmentation signals were confined strictly to the background model and were notably absent from the vessel or optic disc maps. This clean anatomical compartmentalization demonstrates that the UOFE framework effectively dissects the genetic architecture of the eye, resolving fine-grained associations that are typically obscured in ‘whole-image’ or global embedding analyses.

To validate the biological relevance of the extracted IDFs and demonstrate the distinct utility of each anatomical stream, we employed Linkage Disequilibrium Score Regression (LDSC). This analysis revealed a striking double dissociation in genetic correlations (Fig F and Table M in the S3 File), perfectly aligning our unsupervised structural decomposition with known clinical pathology. Specifically, Glaucoma exhibited strong, extensive correlations with Optic Disc IDFs (e.g., Latent 31, $r_{g}=\;-\text{0}.\text{3}\text{9}$, $P_{adj}\;<{\text{1}\text{0}}^{-\text{1}\text{1}}$), consistent with its clinical characterization as an optic neuropathy. In contrast, Age-related Macular Degeneration (AMD) showed nominally significant correlations specifically with Background IDFs (e.g., Latent 4, $P_{adj}<\text{0}.\text{0}\text{5}$) and Vessel IDFs, but zero overlap with Optic Disc features, matching its primary pathology in the retinal pigment epithelium and choroid. Furthermore, systemic hypertension displayed significant genetic overlap specifically with Vessel-derived IDFs, supported by the shared signal at the MEF2C locus.

Taken together, these results not only validate the biological basis of the latent features but also clearly illustrate the power of our multi-stream architecture: by disentangling the retina, we can map complex systemic and ocular diseases to their specific anatomical origins.

**Fig 6:**
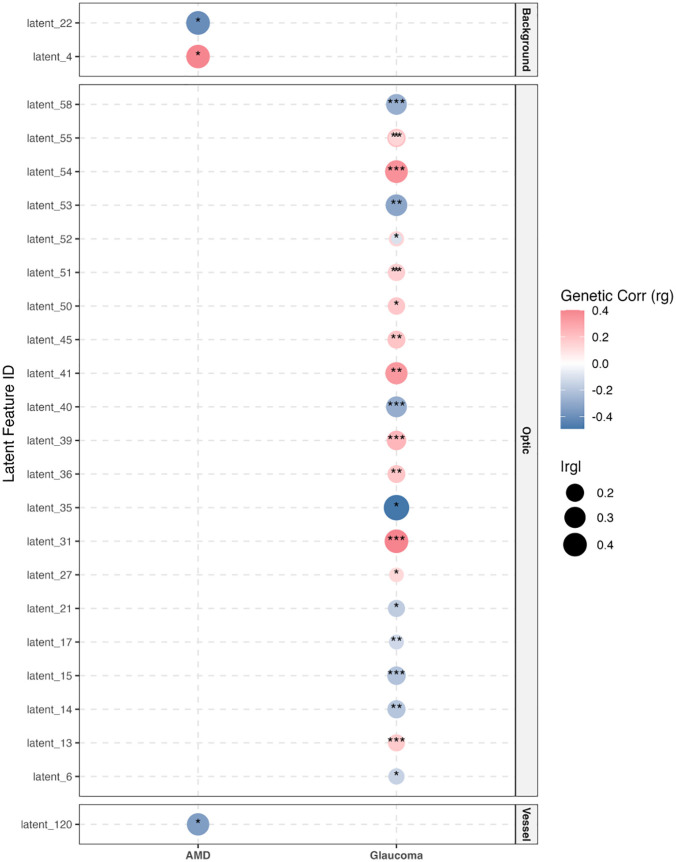
Bubble Heatmap of Genetic Correlation Analysis (LDSC). Notes: The size of the bubbles corresponds to the magnitude of correlation ($r_{g}$), and color indicates direction (Red: positive; Blue: negative). P-values are adjusted for multiple testing using Bonferroni method, Asterisks denote statistical significance (* P_adj_ < 0.05, ** P_adj_ < 0.01, *** P_adj_ < 0.001).

In addition, we validated the biological basis of the latent features via SNP-based heritability ($h^{\text{2}}$) estimates. As detailed in Table 2, the Background IDFs demonstrated the most widespread heritability (up to 76.2% significant), whereas the Cup/Disc IDFs yielded the strongest individual genetic signals (Max $h^{\text{2}}$ = 0.26), reflecting the capture of highly heritable structural markers. The genomic inflation factors ($\lambda_{GC}$) remained close to 1.0 across all categories, confirming that these estimates represent genuine biological variation free from population stratification (Fig 6).

**Table 2:** Summary of Loci-based Heritability Estimates for Latent Features Derived from Retinal Phenotypes.

Category	Eye	N_Features	N_Significant	Pct_Significant	Mean_h2	SE_h2	Max_h2	Mean_Lambda	Mean_Intercept
Background	Left	64	34	0.54	0.043	0.001	0.110	1.030	1.008
Background	Right	64	48	0.76	0.048	0.003	0.112	1.038	1.010
Cup Disc	Left	64	17	027	0.031	0.008	0.260	1.018	1.005
Cup Disc	Right	64	17	027	0.028	0.007	*0.231*	1.017	1.003
Vessel	Left	128	37	029	0.024	0.002	0.158	1.015	1.003
Vessel	Right	128	32	025	0.023	0.002	0.115	1.016	1.004

### Prognostic validity and incremental clinical utility of latent features

**Fig 7:**
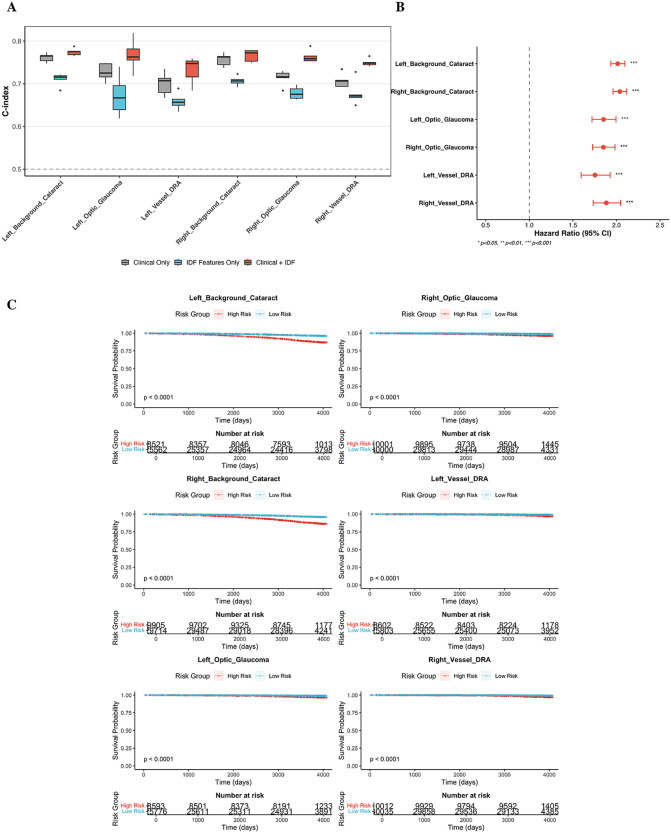
Prognostic Validity and Incremental Clinical Utility of Structure-Specific Latent Features. (Notes: **(a)** Incremental Predictive Performance. Boxplots showing the Concordance Index (C-index) distributions across 5-fold cross-validation for three comparative Cox models: the Clinical Baseline (grey), the structure-specific IDF-only model (blue), and the Combined model (red). **(b)** Prognostic Effect Size of Structure-Specific Latent Features Across Disease Outcomes by using Lasso-penalized Cox proportional hazards ratio model. Forest plot displaying the Hazard Ratio (HR) per 1-standard deviation (SD) increase in the continuous latent risk score. **(c)** Kaplan-Meier Survival Curves Stratified by Structure-Specific Latent Risk Scores(High Risk: top 25%; Low Risk: bottom 75%). Shaded areas represent 95% CI. Log-rank tests confirm significant separation (P < 0.0001) between risk groups across all disease-structure pairs, demonstrating robust clinical stratification capability.

**Robust predictive effect size.** When modeled as a continuous biomarker, the latent risk scores demonstrated substantial predictive power across all phenotypes (P_adj_ <0.001), as illustrated in the forest plot (Fig 7b). The predictive signal was particularly profound in the vascular domain: Vessel-derived IDFs yielded a Hazard Ratio (HR) of 2.38 (95% CI: 2.17–2.61) per standard deviation increase for DRA in the left eye. This indicates that for every SD increase in the vascular anomaly score, the risk of developing diabetic complications effectively doubles—a magnitude of effect that highlights the sensitivity of the model to early microvascular pathology. Similarly, Background and Optic Disc features showed robust associations with cataract and glaucoma (HR ≈ 2.0–2.1), confirming that the IDFs capture a graded, continuous spectrum of disease risk rather than coarse binary categories.

**Incremental predictive value and architectural superiority**. To rigorously quantify the clinical utility of our learned representations, we benchmarked their prognostic performance against standard clinical factors and a monolithic model baseline. All performance metrics (C-index) were evaluated using a 5-fold nested cross-validation framework.

The proposed SA-VAE model yielded consistent and significant improvements in discrimination over the clinical baselines across all phenotypes (Table 3 and Fig 7). For Glaucoma, For Glaucoma, the Optic Disc IDFs provided the most substantial gain, increasing the C-index from a clinical baseline of ~0.714–0.727 to ~0.764–0.767 (ΔC ≈ 0.04–0.05). This net gain is particularly notable given the difficulty of improving upon age-based models in chronic disease prediction. In the vascular domain, Vessel IDFs significantly improved the prediction of Diabetic Retinal Abnormalities (DRA), raising the C-index from ~0.700–0.707 to ~0.732–0.750 (ΔC ≈ 0.03–0.04).

**Table 3:** Summary of Incremental Prognostic Performance for Ocular and Systemic Diseases Derived from 5-Fold Cross-Validation.

Disease Outcome	Clinical Baseline C-index	Structure-Specific C-index	Combined Model C-index	Improvement (ΔC)	Hazard Ratio (95% CI)	P Value
Left_Background_Cataract	0.761 ± 0.01	0.71 ± 0.015	0.774 ± 0.009	0.013	2.01(1.94-2.1)	<0.001
Right_Background_Cataract	0.756 ± 0.015	0.707 ± 0.011	0.766 ± 0.014	0.01	2.04(1.96-2.12)	<0.001
Left_Optic_Glaucoma	0.727 ± 0.021	0.672 ± 0.048	0.767 ± 0.037	0.04	1.85(1.72-1.99)	<0.001
Right_Optic_Glaucoma	0.714 ± 0.018	0.678 ± 0.015	0.764 ± 0.015	0.05	1.85(1.73-1.98)	<0.001
Left_Vessel_DRA	0.7 ± 0.027	0.659 ± 0.02	0.732 ± 0.031	0.032	1.75(1.59-1.93)	<0.001
Right_Vessel_DRA	0.707 ± 0.017	0.678 ± 0.029	0.75 ± 0.009	0.043	1.88(1.73-2.05)	<0.001

Notes: C-index values are presented as Mean ± Standard Deviation across 5-fold cross-validation. Clinical Baseline C-index: Baseline model including age, sex, BMI, smoking status, and alcohol consumption. Structure-Specific C-index: Model including only selected latent features. Combined Model C-index: Integrated model with both clinical covariates and latent features. Improvement(ΔC): Represents the net improvement in C-index (Combined minus Clinical). P Values are derived from Lasso-penalized Cox proportional hazards ratio model.

Even for Cataracts, against a very high age-dependent clinical baseline (~0.76), Background IDFs conferred a statistically significant performance boost (ΔC ≈ 0.01, final C-index ~0.77), likely by capturing subtle signals of fundus haziness not encoded in age alone.

Crucially, comparison with the Monolithic Baseline confirmed the necessity of our structure-aware strategy. As detailed in Table 3, the proposed SA-VAE model consistently and significantly outperformed the monolithic approach across all disease outcomes (paired t-test, P < 0.05). This confirms that explicit anatomical disentanglement extracts cleaner, more predictive biological signals than holistic, unsegmented image embeddings.

**Risk stratification capability**. Kaplan-Meier analysis (Fig 7c) further validated the clinical applicability of these risk scores. Survival trajectories for the high-risk group (top 25%) diverged significantly from the low-risk group across all cohorts (P < 0.0001). Crucially, this separation was highly concordant between left and right eyes, reinforcing the biological robustness of the features. For DRA, despite a lower baseline incidence compared to cataracts, the vessel-specific model successfully identified a high-risk subpopulation with a markedly accelerated disease trajectory. Collectively, these findings demonstrate that UOFE-derived features provide stable, clinically meaningful risk gradients that refine prognosis beyond conventional risk factors.

## Discussion

In this study, we introduced UOFE, a structure-aware deep learning framework designed to overcome the interpretability and sensitivity bottlenecks inherent in traditional “black-box” retinal analysis. By explicitly disentangling the fundus image into biologically distinct components—vasculature, optic disc, and background—our approach not only achieved high-fidelity phenotypic extraction but also significantly amplified the genetic discovery yield compared to monolithic baselines.

A central insight from our work is that phenotypic resolution dictates genetic power. Monolithic models often suffer from a “masking effect,” where dominant anatomical features (e.g., the optic disc) suppress subtle signals from peripheral capillaries or background textures. Our comparative analysis confirms that UOFE effectively rescues these submerged signals. By enforcing a “divide-and-conquer” strategy that learns independent latent spaces for each substructure, we maximized the signal-to-noise ratio, enabling the discovery of thousands of unique loci that were effectively invisible to standard whole-image approaches.

A critical validation of our unsupervised approach lies in the biological interpretability of the learned features. Addressing concerns regarding the potential entanglement of overlapping structures (e.g., vessels crossing the optic disc), our LDSC analysis revealed a striking genetic double dissociation: glaucoma genetics mapped primarily driven by Optic Disc features, while AMD genetics [19–21] mapped specifically to Background features. This clean compartmentalization demonstrates that the model successfully disentangled neurodegenerative from metabolic/pigmentary pathologies in the latent space, without relying on supervised disease labels. Furthermore, despite the inherent challenges in reconstructing sparse vascular networks (reflected in pixel-wise metrics like SSIM), the biological fidelity of our Vessel model is robustly supported by its specific mapping to angiogenesis pathways (e.g., MEF2C, FGF5) and its exclusive association with Systemic hypertension. This reinforces the retina-as-a-window hypothesis by identifying shared genetic drivers of retinal vascular caliber and systemic circulatory health.

Beyond genetic discovery, Beyond genetic discovery, the UOFE framework demonstrates robust translational utility for disease risk prediction (Table 3). Prior imaging-based risk models typically rely on subjective, manually engineered markers or ‘black-box’ monolithic deep learning features that lack anatomical specificity. In our strictly controlled, 5-fold cross-validated benchmarking, the addition of structure-specific SA-VAE features yielded substantial incremental prognostic value over standard clinical covariates (e.g., ΔC ≈ 0.05 for Glaucoma). Crucially, the SA-VAE systematically outperformed the monolithic baseline across all evaluated diseases. This confirms that explicitly encoding distinct structural domains—such as vascular topology for diabetic complications or optic disc geometry for glaucoma—prevents these critical high-frequency signals from being obscured by the global background. Consequently, these disentangled latent scores function as highly sensitive biological markers (HR ≈ 1.8–2.0 per SD increase), offering a scalable, interpretable tool for disease risk stratification in large population cohorts..

This study has several limitations. First, it is important to contextualize our unsupervised genetic discovery power relative to traditional, hypothesis-driven GWAS. Supervised models trained on strictly defined clinical metrics naturally yield higher statistical power for those isolated traits. This represents an expected trade-off. While our SA-VAE compresses the entire complex morphology of an anatomical region into a composite representation, which may dilute the genetic signal of any single traditional metric, its overarching advantage lies in hypothesis-free global discovery. This unbiased approach uniquely uncovers novel associations across unannotated microvascular and background structures that supervised algorithms inherently ignore. Second, while our CoxPH models demonstrated strong prognostic utility for UOFE embeddings, we lacked direct benchmarking against the monolithic ViT or supervised baselines in a survival context. Future research should formally quantify this incremental predictive value and explore (semi-)supervised learning paradigms—such as guiding the latent space with disease labels—to potentially yield further performance improvements. Thirdly, regarding the macular region, our framework adopted an implicit encoding strategy within the background model rather than an explicit structural segmentation. While this approach effectively captures photometric phenotypes (e.g., pigment density) highly relevant to AMD genetics (TYR, HERC2) [22], it limits the direct quantification of geometric foveal metrics, such as the foveal avascular zone. Future iterations could incorporate dedicated foveal attention modules to explicitly resolve these fine-grained boundaries. Finally, while our models were validated across diverse external imaging datasets, the primary genetic discovery and prognostic evaluations were based on European ancestry. Large-scale, cross-ancestry replication remains essential to ensure the global generalizability of these structure-specific genetic architectures.

## Conclusion

UOFE represents a paradigm shift from monolithic, black-box representations towards structure-aware AI in ophthalmology. By providing a scalable, interpretable, and genetically validated framework that disentangles complex anatomical architectures, this tool paves the way for next-generation digital biomarker discovery and precision risk stratification.

## Materials and methods

### Overview of the UOFE framework

The Unsupervised Ophthalmic Feature Extraction (UOFE) framework (see Fig 1) was designed as a pipeline to identify novel, “data-driven” biomarkers by disentangling fine-grained structural features isolating the optic disc/cup, vasculature, and retinal background, and to establish their association with ocular and systemic disease and genetic architecture. It proceeds in three stages: (1) Structure-Aware Feature Encoding, where raw images are disentangled into vessel, disc/cup, and background components; (2) Multi-Dimensional Validation, utilizing quantitative metrics and visual perturbation to ensure feature interpretability; and (3)Translational Application, deploying extracted features for ocular and systemic disease prediction and genetic discovery.

1. **Data resource and study population**

The EyePACS dataset [15] served as the primary resource for model training. After strict quality control [23], 53,600 high-quality images were retained. Crucially, to prevent data leakage, the dataset was split into training (75%, N = 40,200) and internal validation (25%, N = 13,400) sets at the participant level, ensuring that images from the same individual remained within the same partition.

For large-scale feature extraction and genetic discovery, we processed 130,329 images from the UK Biobank (UKB) cohort [Project ID: 151140)]. Our genetic analysis focused on 75,010 UKB participants with paired high-quality imaging and genotype data. (Category 100315). To evaluate demographic robustness, we further conducted subgroup analyses comparing White and non-White populations. To evaluate model generalizability, we constructed an independent external test set by randomly sampling 1,000 images from a diverse pool of approximately 170,000 images across 15 public datasets (Table A in S3 File).

1. **Stage 1: Structure-aware feature encoding**

### Imaging preprocessing and structural segmentation

Initial QC for RF images from EyePACS, UKB and 15 public validation datasets was conducted using Swin-MCSFNet12 [23].

To operationalize our structure-aware strategy, we utilized the Automorph module [24] to generate precise binary masks for specific morphological structures: the optic disc/cup and retinal blood vessels. Our approach feeds the full fundus images, optic disc masks, and vessel masks into three isolated structure-aware autoencoders. Crucially, these streams share no parameters and are optimized separately to explicitly encode the macular background, optic disc, and retinal vasculature.

To ensure the background stream specifically captures the retina’s textural and pigmentary landscape, we applied a 17 × 17 median smoothing kernel to the full fundus images [14]. This kernel intentionally attenuates high-frequency vascular details while preserving low-frequency variations inherent to global illumination and pigment density. Although macroscopic landmarks (e.g., the optic disc and fovea) remain discernible as luminosity gradients, this filtering successfully isolates the retina’s photometric profile from sharp geometric boundaries. Ultimately, this ensures that texture learning is decoupled from structural constraints, forcing each isolated stream to capture strictly non-redundant biological information.

### Structure-specific VAE architecture and monolithic VAE baseline

To encode these disentangled inputs, we designed the SA-VAE comprising three independent VAE streams: VAE_vessel_, the VAE_disc-cup_, and VAE_background_. All streams shared a foundational convolutional architecture consisting of a 5-layer encoder and a 5-layer decoder(detailed in Method in S1 File). However, domain-specific modifications were implemented to optimize feature extraction for each substructure. For instance, the VAE_vessel_ model incorporates a sharpness-aware loss term and encoding-stage dropout layers to mitigate overfitting to sparse binary vascular masks.

To evaluate the performance of the SA-VAE model, we trained a monolithic VAE baseline (VAE_Monolithic_) on raw, whole fundus images. The VAE_Monolithic_ utilizes the same encoder-decoder backbone and latent space dimensionality as the UOFE streams but optimizes a global reconstruction objective. This baseline serves as a control to assess the model’s ability to capture fine-grained phenotypes (e.g., vascular details) without explicit structural decomposition.

### Model training and dimensionality selection

Models were trained on the EyePACS dataset using an NVIDIA 3090Ti GPU, We optimized the standard Variational Autoencoder objective function, which minimizes the combination of the Mean Squared Error (MSE) for image reconstruction and the Kullback-Leibler (KL) divergence to regularize the latent space distribution.

We determined the empirically selected latent dimensionality ($d^{*}$) for each substructure rather than arbitrarily. By performing a grid search d ∈ {8,16,32,64,128,256}, we selected dimensions that corresponded to the saturation point of reconstruction quality on the validation set, thereby balancing model compactness with information retention. The final dimensions were set as: $\overset{*}{\underset{background}{d}}=\text{64}$, $\overset{*}{\underset{disc\_cup}{d}}=\text{64}$, and $\overset{*}{\underset{vessel}{d}}=\text{128}$ (see Fig 1–6 in S2 File).

### Independent feature extraction

We extracted three distinct sets of latent sub-models and concatenated them to form three latent features: $Z_{background\;}\in \;R^{\text{6}\text{4}},Z_{disc\_cup\;}\in \;R^{\text{6}\text{4}}$, and, $Z_{vessel}\in \;R^{\text{1}\text{2}\text{8}}$. These structure-specific sets are collectively referred to as Image-Derived Features (IDFs).

1. **Stage II: Multi-dimensional validation of latent features**

### Quantitative evaluation: Disentanglement and reconstruction

To verify that UOFE effectively disentangles distinct retinal phenotypes, we computed rigorous quantitative metrics on the UK Biobank set. Given the inherent physiological correlations among retinal traits, we first performed Principal Component Analysis (PCA) on the ground-truth morphological metrics to derive orthogonal axes of variation [25].We calculated Disentanglement, Completeness, and Informativeness (DCI) scores [26] to evaluate the exclusivity of the mapping between latent dimensions and true biological factors, averaging the results across 10 independent resampling iterations (N = 1,000 per iteration) to ensure robustness; and (2) the Mutual Information Gap (MIG) [27], which quantifies the compactness of the feature representation.

Additionally, reconstruction quality was evaluated using SSIM, PSNR, and LPIPS across the 15 public validation sets.

### Qualitative assessment of feature interpretability

Complementing our quantitative metrics, we conducted latent space perturbation experiments to qualitatively decode the biological semantics of individual IDFs. We systematically traversed each latent dimension $z_{i}$ across the range ${[z}_{i}-\text{3}\sigma ,\;z_{i}+\text{3}\sigma ]$ while holding other dimensions constant. The resulting reconstructions were analyzed by generating difference maps (t-maps), which spatially highlight the specific anatomical regions modulated by each feature. This approach allows for the direct mapping of abstract latent features to localized structural variations in the retina.

1. **Stage III: Downstream clinical and genetic applications**

### Ocular and systemic disease prediction

To evaluate the incremental clinical utility of the learned representations, we modeled the risk of major ocular and systemic outcomes (glaucoma, cataract, and diabetic retinal abnormalities) using Cox proportional hazards models. Models were run separately for the left and right eyes and adjusted for age, sex, BMI, systolic blood pressure, and type 2 diabetes status.

Simtaneusly, we employed a 5-fold nested cross-validation framework. Within each iteration, a Lasso-penalized Cox model was trained and hyperparameter-optimized on the training folds. The resulting model was then applied to the held-out test fold to generate unbiased, out-of-sample risk scores. Predictive performance was evaluated solely on these out-of-sample scores using the Concordance Index (C-index), Hazard Ratios (HR) per standard deviation increase, and Kaplan-Meier survival stratification.

### Genetic association and architecture analysis

First, we estimated SNP-based heritability ($\overset{\text{2}}{\underset{SNP}{h}}$) for each latent feature using GCTA [28] to confirm that the extracted IDFs capture heritable biological variation rather than imaging noise. Then, we performed single-trait GWAS on the extracted IDFs using the fastGWA tool (GCTA) [28], genetically inferred ancestry using the top 10 principal components (PCs) provided by the UK Biobank, alongside age, sex, and genotyping array. To improve statistical power and capture pleiotropic effects across correlated latent features, we employed a multi-trait GWAS framework (C-GWAS) [13].

To evaluate the full UOFE framework comprehensively, the individual latent vectors extracted from the three independent SA-VAE streams were first concatenated into a single, unified feature representation per participant prior to association testing (see Fig 4). Conversely, for stream-specific analyses (see Fig 5), the latent vector from each respective anatomical stream was evaluated independently within the C-GWAS pipeline. Finally, to validate the biological relevance of our IDFs beyond locus discovery, we utilized Linkage Disequilibrium Score Regression (LDSC) to estimate genetic correlations ($r_{g}$) with established clinical phenotypes (e.g., Glaucoma, AMD). This analysis determines whether the genetic architecture of our unsupervised features specifically overlaps with known disease biology, providing a robust, independent validation of their functional significance.

## Supporting information

S1 File Methods. (DOCX)

S2 File Figures. Fig A. Comparison of genome-wide significant independent loci identified by UOFE and iGWAS. Fig B–Fig G. Latent Features Distribution. Fig H–Fig J. Results of Perturbation Experiment. Fig K–Fig P. Results of Quantitative Analysis on Three Substructures. Fig Q–Fig S. Results of Latent Features Heritability Analysis. Fig T. SNP-based Heritability of Latent Features on Three Substructures. Fig U. Results of GO Enrichment Analysis.
(DOCX)

S3 File Tables. Table A. 15 External Validation Dataset Information. Table B. Comparison of Reconstruction Fidelity Between the Proposed SA-VAE Framework and the Monolithic Baseline. Table C. Quantitative Evaluation of Disentangled Latent Representations. Table D–Table L. Summary of C-GWAS Loci. Table M. Significant Results of LDSC Analysis.
(DOCX)
