# Supplementary material for: Structure-aware retinal disentanglement reveals the genetic architecture of ocular and systemic diseases
Source: PLOS Digit Health. 2026 May 15;5(5):e0001376. doi: 10.1371/journal.pdig.0001376 (PMC13178874; doi:10.1371/journal.pdig.0001376)
Supplement: S1 File — (DOCX) [file pdig.0001376.s001.docx]

**Structure-Aware Retinal Disentanglement Reveals the Genetic Architecture of Ocular and Systemic Diseases**

**Methods**

**Contents:**

1.1. Genetic Data Preprocessing

1.2. RF Imaging Preprocessing

1.3. VAE-Optic Model Design and Parameter Optimization for Retinal Feature Extraction

1.4. Two-Stage VAE-Optic Model Training and Performance Evaluation

1.5. Latent IDFs Extraction

1.6. Biological Relevance Validation of Latent IDFs: Perturbation Analysis

1.7. Exploring Feature-Disease Relevance: Latent IDF-Based Disease Prediction

1.8. Substructure-Specific Genetic Associations and Systemic pleiotropy

1.9. Detailed Configuration of the Monolithic Vision Transformer (ViT) Baseline

Reference

**1.1 Genetic Data Preprocessing**

Genetic data underwent several preprocessing steps to ensure quality and accuracy^[1]^ (see Fig. 1 in the Supplementary Materials). These included: Pre-imputation quality control (QC) to ensure clean data; Phasing was performed using SHAPEIT2^[2]^, and genotype imputation was carried out using Minimac4 with the HRC reference panel on the Michigan Imputation Server^[3]^; Post-imputation QC involved excluding single nucleotide polymorphisms (SNPs) with an imputation quality score (R²) below 0.8 using BCFTOOLS^[4]^.

These steps ensured that the genetic data were high-quality and suitable for downstream analysis.

**1.2 RF Imaging Preprocessing**

All RF images from both the EyePACS dataset and the UK Biobank underwent initial QC using the Swin-MCSFNet12 model^[5]^. Following image preprocessing, including removal of peripheral black borders, resizing, and normalization, the model evaluated image quality through multi–color-space feature fusion based on RGB, HSV, and LAB representations. Using a Swin-Transformer backbone, images were classified into three quality categories: good, usable, and reject. Images graded as reject were excluded from subsequent analyses. Following QC, the Automorph segmentation module^[6]^ was utilized to segment the optic cup-disc and retinal vessel regions. A 17×17 median smoothing kernel was applied to the images during preprocessing to enhance the background-only images. This kernel size was chosen based on the anatomical features, as retinal blood vessels typically have diameters smaller than 17 pixels ^[7,8]^.

**1.3 VAE-Optic Model Design and Parameter Optimization for Retinal Feature Extraction**

We developed three variational autoencoder (VAE)-Optics models to capture low-dimensional representations from distinct subregions: retinal vessel, optic disc/cup, and background regions(detailed in Supplementary Material 1). Each model utilizes a convolutional encoder-decoder architecture, designed to learn abstract representations of retinal structures. The encoder consists of five convolutional layers, each followed by a ReLU activation function to introduce non-linearity. These layers progressively downsample the input image $x$ from EyePACS dataset, reducing its spatial dimensions while increasing the number of feature channels. The output of the encoder is then flattened and mapped into a latent space via two parallel fully connected layers, which output the parameters of a Gaussian distribution: the mean vector $\mu$ and the log-variance vector $\log\sigma^{2}$, computed as：

$\mu=f_{mean}\left( Flatten\left( Encoder\left( x \right) \right) \right)$ and$\log\sigma^{2}= f_{logvar}\left( Flatten\left( Encoder\left( x \right) \right) \right)$,

where $x$ represents the input image of the respective retinal sub-structures.

A latent vector $z \in R^{d}$ is then sampled using the standard reparameterization trick:

$z=\mu+\sigma\cdot\varepsilon$，

where $\varepsilon\sim N\left( 0,1 \right)$, and $\sigma$is obtained by exponentiating half the log-variance. This formulation ensures differentiability during training while allowing stochastic sampling of the latent space.

The decoder reconstructs the original high-dimensional image $\hat{x}$ from the latent vector $z$ through a series of transposed convolutional layers:

$\hat{x}$ = $f_{decoder}\left( z \right)$

Each transposed convolutional layer is followed by a ReLU activation function, except for the final layer, which employs a Tanh activation to normalize the output to the same range and resolution as the input image. This architecture ensures that the latent vector $z$ captures essential structural information of each retinal region, facilitating precise reconstruction and interpretation.

For the retinal vessel model, we introduced two key modifications to address the inherent challenges of working with the complex nature of binary vascular masks, particularly overfitting. To improve the model's generalization ability and prevent overfitting, the input binary vascular images were pre-processed using data augmentation techniques, such as random horizontal flipping, random rotations, and color jittering. These augmentations helped create a more diverse training set, allowing the model to capture a broader range of vascular features. Additionally, a dropout function was incorporated during the downsampling process of the VAE-Optics model. This technique randomly deactivates neurons during training, preventing overfitting by promoting better generalization.

**1.4 Two-Stage VAE-Optic Model Training and Performance Evaluation**

The models were trained using a two-stage strategy to ensure both high-fidelity reconstruction and a well-regularized latent space. In the first stage, each model was trained for 50 epochs with varying latent dimensions $d \in\left\{ 8+2k | k \in N, d \leq256 \right\}$. For each configuration, the validation loss $\mathcal{\mathcal{L}}\left( x \right)$ was computed, and the optimal dimension $d^{*}$ was determined as:

$$d^{*}=\arg\min_{d} \mathcal{\mathcal{L}}\left( x;d \right)$$

In the second stage, the model was retrained from scratch for 200 epochs using the selected optimal latent dimension $d^{*}$.To ensure high-fidelity reconstruction and a well-regularized latent space, the models were trained to minimize the standard VAE loss function $\mathcal{\mathcal{L}}\left( x \right)$, defined as:

$$\mathcal{\mathcal{L}}\left( x \right)= E_{\mathcal{q}\left( z | x \right)}\left[ logp\left( x | z \right) \right]-D_{\mathrm{KL}}\left( q\left( z | x \right)||p\left( z \right) \right)$$

Where $\mathcal{q}\left( z | x \right)$ is the approximate posterior distribution parameterized by the encoder, $p\left( z \right)$ is the prior distribution over the latent variable $z$, and $p\left( x | z \right)$ is the likelihood of reconstructing the input image $x$ from the latent representation $z$, parameterized by the decoder. This loss function balances reconstruction fidelity and latent space regularization, forming the basis of variational inference in the VAE framework.

Training was conducted on an NVIDIA 3090Ti GPU, ensuring efficient computation. The best-performing model on the validation set was saved for downstream feature extraction.

**1.5 Latent IDFs Extraction**

We applied the trained VAE-Optical models toto extract latent features from each segmented retinal subregion. These features were represented as $Z_{background}(n\times d_{background}^{*})$, $Z_{disc\_cup}\left( n\times d_{disc\_cup}^{*} \right)$, and $Z_{vessel}\left( n\times d_{vessel}^{*} \right)$, where n represents the number of participants and $d$ denotes the dimensionality of the latent feature space for each specific retinal region.

**Biological Interpretability: Validation through Perturbation Experiments and Systemic Disease Prediction**

To assess the statistical significance and biological interpretability of the latent features, we performed perturbation experiments and evaluated the predictive power of these features in the context of systemic disease outcomes.

- 1. **Biological Relevance Validation of Latent IDFs: Perturbation Analysis**

To evaluate the biological relevance of specific latent IDFs with morphological variations in retinal vessels, optic disc/cup size, and background structures, we performed a perturbation analysis to assess the robustness and relevance of the latent representations by systemically adding Gaussian noise to the latent variables. For each selected IDF, perturbations were introduced by adding noise proportional to its standard deviation (i.e., $z^{ʻ}=z+ \propto\cdot\sigma\cdot\varepsilon,$where $\varepsilon\sim N\left( 0,1 \right)$), and $\propto$is a scaling factor), and the corresponding changes in reconstructed images were examined. This approach enabled us to interpret the morphological sensitivity of each latent feature. Using retinal vessels as an example, we randomly divided $Z_{vessel}$ into 5 groups to assess the robustness of the perturbation effects. For each group, $Z_{vessel}$ was first decoded into reconstructed outputs ($\hat{x}$), and then perturbed by adding $a$magnitude of $3\cdot\sigma_{Z_{vessel}}$, resulting in a perturbed perturbation $z_{vessel}^{ʻ}$($=Z_{vessel}+3\cdot\sigma_{Z_{vessel}}$) , which was subsequently decoded into perturbed reconstructed outputs, ($\hat{x^{ʻ}}$). To enhance the contrast between $\hat{x} and \hat{x^{ʻ}}$, we introduced a Sharpening Vessel-Variation Layer (SVVLayer), which applies a 9×9 convolutional sharpening kernel to both $\hat{x}$and $\hat{x^{ʻ}}$. This operation accentuates vascular structures, improving the detection of perturbation-induced morphological differences. Additionally, a randomly sampled weighting factor(W) from a uniform probability distribution (W∼U(0.2,0.4)) was incorporated to modulate vessel morphology in the generated images. This adjustment not only introduces biologically realistic variations in vessel patterns but also mitigates the risk of generating overly uniform or anatomically implausible structures. Next, we performed paired t-tests between $\hat{x} and \hat{x^{ʻ}}$ in each of the 5 groups to calculate the t-value matrix (t-map). The resulting t-map was smoothed using Gaussian filtering and normalized before being overlaid onto normal fundus images, providing a visual representation of the regions influenced by specific latent dimensions. Furthermore, the mean and standard deviation of the smoothed t-values for each $Z_{vessel}$ were calculated to evaluate the stability and importance of each $Z_{vessel}$ in the perturbation analysis of latent space.

- 1. **Exploring Feature-Disease Relevance: Latent IDF-Based Disease Prediction**

To assess the association between latent features and disease risk, we employed survival analysis techniques. Specifically, we utilized Cox proportional hazards (CoxPH) regression models to investigate whether the latent variables could identify high-risk individuals for disorders of refraction and accommodation (DRA), glaucoma, and cataracts. The analyses were conducted separately for the left and right eyes. Time-to-event data were defined using the date of the first RF imaging recorded at enrollment in the UK Biobank as the starting point. Participants were followed until January 2022 or censored at the end of the follow-up period. The CoxPH models were adjusted for potential confounders, including relevant demographic and clinical covariates such as age, sex, BMI, smoking status, alcohol consumption, and others. To assess the discriminatory ability of the latent features in predicting disease outcomes, the concordance index (c-index) was computed.

- 1. **Substructure-Specific Genetic Associations and Systemic pleiotropy**

We performed single-trait GWAS using the fastGWA^[9]14]^ function (v1.94.1) implemented in GCTA on a discovery cohort of native English speakers from the UK Biobank. The phenotypes used were latent variables ($Z_{background}$, $Z_{vessel}$, and $Z_{disc\_cup}$) derived from RF images via three distinct VAE-Optics models, each specialized for one anatomical subregion: background, blood vessels, and optic disc/cup.

Before performing single-trait GWAS, we computed sparse genetic relationship matrices from the corresponding imputed genotype data to account for relatedness and population structure. The distributions of the latent features were evaluated and confirmed to approximate normality. To mitigate the influence of confounding factors, each latent phenotype was adjusted for sex, BMI, smoking status, alcohol consumption, and the top 10 genetic principal components (PCs).

To explore the shared genetic architecture underlying the three latent IDFs, we conducted multi-trait GWAS, like C-GWAS. This method combines the summary statistics from single-trait GWAS to decompose trait correlations into two components: (i) genetic correlations due to pleiotropic genetic variants, and (ii) background correlations arising from shared environmental or technical factors. We also validated the C-GWAS findings in an independent replication cohort composed of native and non-native English speakers. A locus was considered successfully replicated if its lead variant achieved significance (P-value < 0.05/M_v_), where M_v_ represents the number of approximately independent variants tested after linkage disequilibrium (LD) pruning.

For functional interpretation, we employed the FUMA (v1.6.0) pipeline to map C-GWAS-identified loci. Genomic risk loci were defined using a LD threshold of r² ≥ 0.6 based on the 1000 Genomes Phase 3 European reference panel. Independent lead single-nucleotide polymorphisms (SNPs) were identified using stricter pruning (r² < 0.1) to ensure non-redundancy.

To further annotate and functionally interpret the lead variants, we used GWASLab^[10]^ to assess whether the identified variants fell within ±250 kb of previously reported genome-wide significant SNPs associated with systemic or ophthalmic traits. This analysis aimed to identify regions of genetic colocalization or pleiotropy between the latent retinal features and known disease-relevant loci.

**1.9. Detailed Configuration of the Monolithic Vision Transformer (ViT) Baseline**

To ensure a fair and rigorous evaluation of the UOFE framework, we implemented a monolithic Vision Transformer (ViT) as the primary baseline for retinal embedding and subsequent downstream genetic discovery. This monolithic baseline was designed as a strong, state-of-the-art single-stream comparator rather than a strictly component-matched ablation of our proposed SA-VAE. By benchmarking against a ViT, we aimed to contextualize our framework's performance against a high-capacity, contemporary standard that lacks explicit anatomical separation. The benchmarking was conducted under strictly matched experimental conditions to isolate the performance gains attributable to our proposed methodology.

We employed a standard ViT architecture (specifically, ViT-B/16) pre-trained on ImageNet-21k, which is widely adopted as a strong baseline for medical image analysis. For downstream GWAS analyses, we utilized the feature representations extracted from the final layer of the network.

Specifically, the128-dimensional from the class tokenwas extracted as the global representation of the retinal image for each participant. Crucially, the monolithic ViT was trained and evaluated using the exact same data splits and preprocessing procedures as UOFE. Retinal images from the UK Biobank were standardized (e.g., center-cropped and resized to 224×224 pixels) before being fed into the network. The model was fine-tuned using the same optimization strategy, including the AdamW optimizer, a learning rate of (1 × 10^-5 ,^ and a batch size of 32, over 200 epochs. Data augmentation techniques (e.g., random horizontal flipping and cropping) were identical across both frameworks.

Following the extraction of the monolithic ViT embeddings, the downstream genetic association analyses were performed utilizing the exact same pipeline applied to UOFE. We adjusted for identical covariates (e.g., age, sex, genotyping array, and the first 10 genetic principal components) using PLINK 2.0. This matched setup ensures that any differences in the number of genome-wide significant loci or lead SNPs discovered are robustly comparable.

Finally, regarding image reconstruction, it is important to acknowledge that the ViT baseline and our proposed SA-VAE utilize fundamentally different reconstruction objectives. Consequently, the superior reconstruction fidelity observed in our results is framed as an advantage of the holistic SA-VAE framework—encompassing both the anatomical compartmentation strategy and the generative VAE objective—rather than being attributed solely to anatomical separation.

**Reference**

[1] Xiong, W. *et al.* Rare Variant Analysis and Molecular Dynamics Simulation in Alzheimer’s Disease Identifies Exonic Variants in FLG. *Genes* **13**, 838 (2022).

[2] Delaneau, O., Marchini, J. & Zagury, J.-F. A linear complexity phasing method for thousands of genomes. *Nature methods* **9**, 179-181 (2012).

[3] Das, S. *et al.* Next-generation genotype imputation service and methods. *Nature genetics* **48**, 1284-1287 (2016).

[4] Danecek, P. *et al.* Twelve years of SAMtools and BCFtools. *Gigascience* **10**, giab008 (2021).

[5] Huang, C. *et al.* Enhancing Retinal Fundus Image Quality Assessment With Swin-Transformer–Based Learning Across Multiple Color-Spaces. *Translational Vision Science & Technology* **13**, 8-8 (2024).

[6] Zhou, Y. *et al.* AutoMorph: automated retinal vascular morphology quantification via a deep learning pipeline. *Translational vision science & technology* **11**, 12-12 (2022).

[7] Saeed, A. Q., Sheikh Abdullah, S. N. H., Che-Hamzah, J., Abdul Ghani, A. T. & Abu-Ain, W. A. K. Synthesizing retinal images using end-to-end vaes-gan pipeline-based sharpening and varying layer. *Multimedia Tools and Applications* **83**, 1283-1307 (2024).

[8] Li, W. *et al.* Generating fundus fluorescence angiography images from structure fundus images using generative adversarial networks. *arXiv preprint arXiv:2006.10216* (2020).

[9] Jiang, L. *et al.* A resource-efficient tool for mixed model association analysis of large-scale data. *Nature genetics* **51**, 1749-1755 (2019).

[10] He, Y., Koido, M., Shimmori, Y. & Kamatani, Y. GWASLab: a Python package for processing and visualizing GWAS summary statistics. (2023).
