# Supplementary material for: Structure-aware retinal disentanglement reveals the genetic architecture of ocular and systemic diseases
Source: PLOS Digit Health. 2026 May 15;5(5):e0001376. doi: 10.1371/journal.pdig.0001376 (PMC13178874; doi:10.1371/journal.pdig.0001376)
Supplement: S2 File — Fig A. Comparison of genome-wide significant independent loci identified by UOFE and iGWAS. Fig B–Fig G. Latent Features Distribution. Fig H–Fig J. Results of Perturbation Experiment. Fig K–Fig P. Results of Quantitative Analysis on Three Substructures. Fig Q–Fig S. Results of Latent Features Heritability Analysis. Fig T. SNP-based Heritability of Latent Features on Three Substructures. Fig U. Results of GO Enrichment Analysis. (DOCX) [file pdig.0001376.s002.docx]

**Structure-Aware Retinal Disentanglement Reveals the Genetic Architecture of Ocular and Systemic Diseases**

**Figures**

**Contents**

Fig A. Comparison of genome-wide significant independent loci identified by UOFE and iGWAS

Fig B-Fig G. Latent Features Distribution

Fig H-Fig J. Results of Perturbation Experiment

Fig K-Fig P. Results of Quantitative Analysis on Three Substructures

Fig Q–Fig S. Results of Latent Features Heritability Analysis

Fig T. SNP-based Heritability of Latent Features on Three Substructures
Fig U. Results of GO Enrichment Analysis


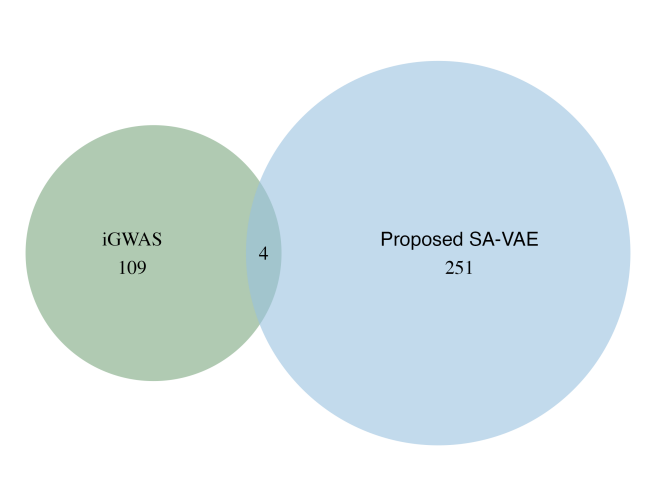


Fig A. Comparison of significant independent loci identified by UOFE and iGWAS

(Notes: The Venn diagram illustrates the overlap of independent genomic loci discovered using retinal embeddings from the proposed UOFE framework compared to the established iGWAS baseline. Both methods were evaluated on the same dataset with identical downstream GWAS pipelines and strict LD clumping (r^2^< 0.1, 250kb window). The proposed SA-VAE identified a total of 255 independent loci, whereas iGWAS identified 113 loci. Notably, only 4 loci were shared between the two approaches.)


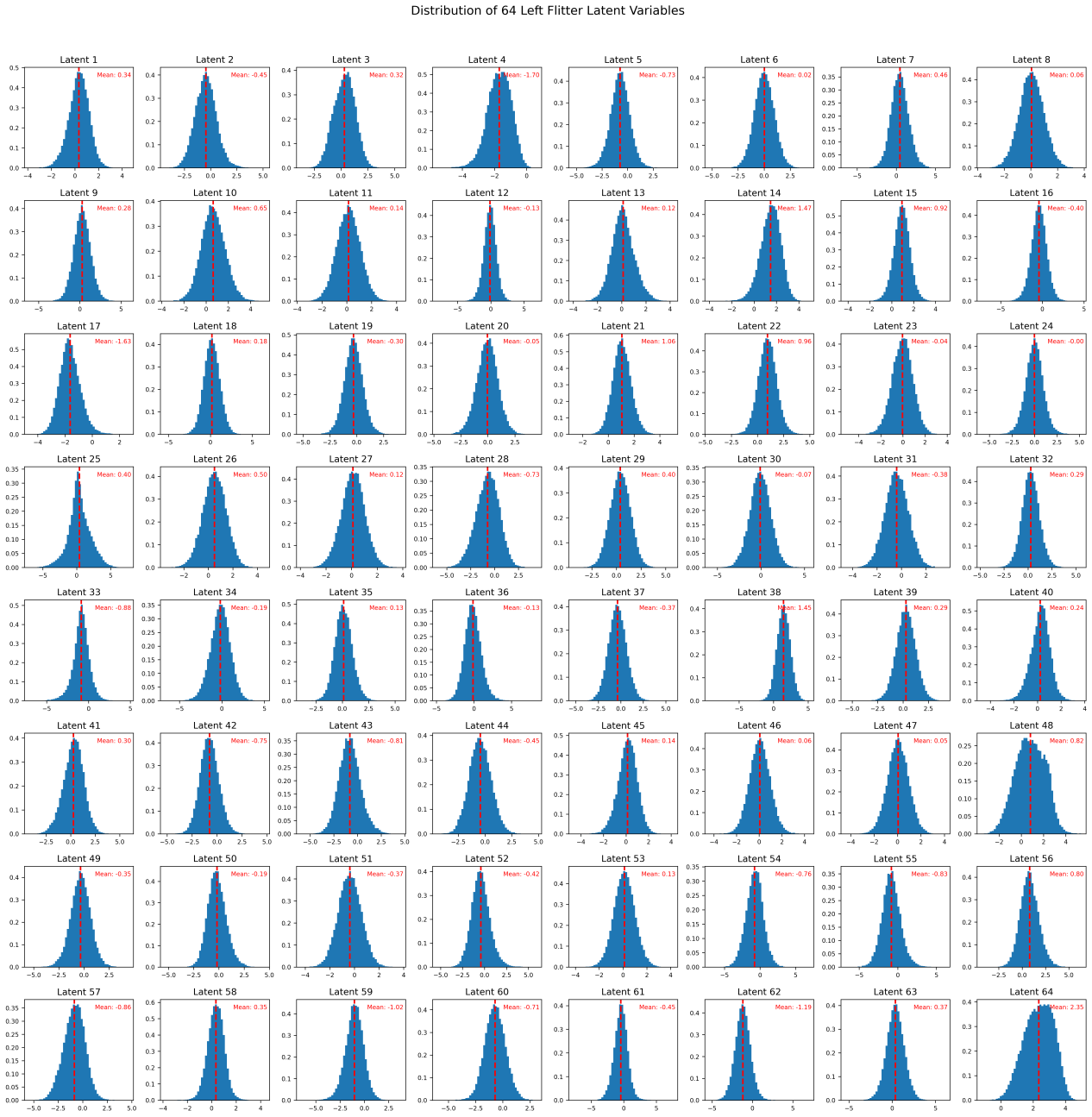
 Fig B. The data distribution of left eye background IDFs


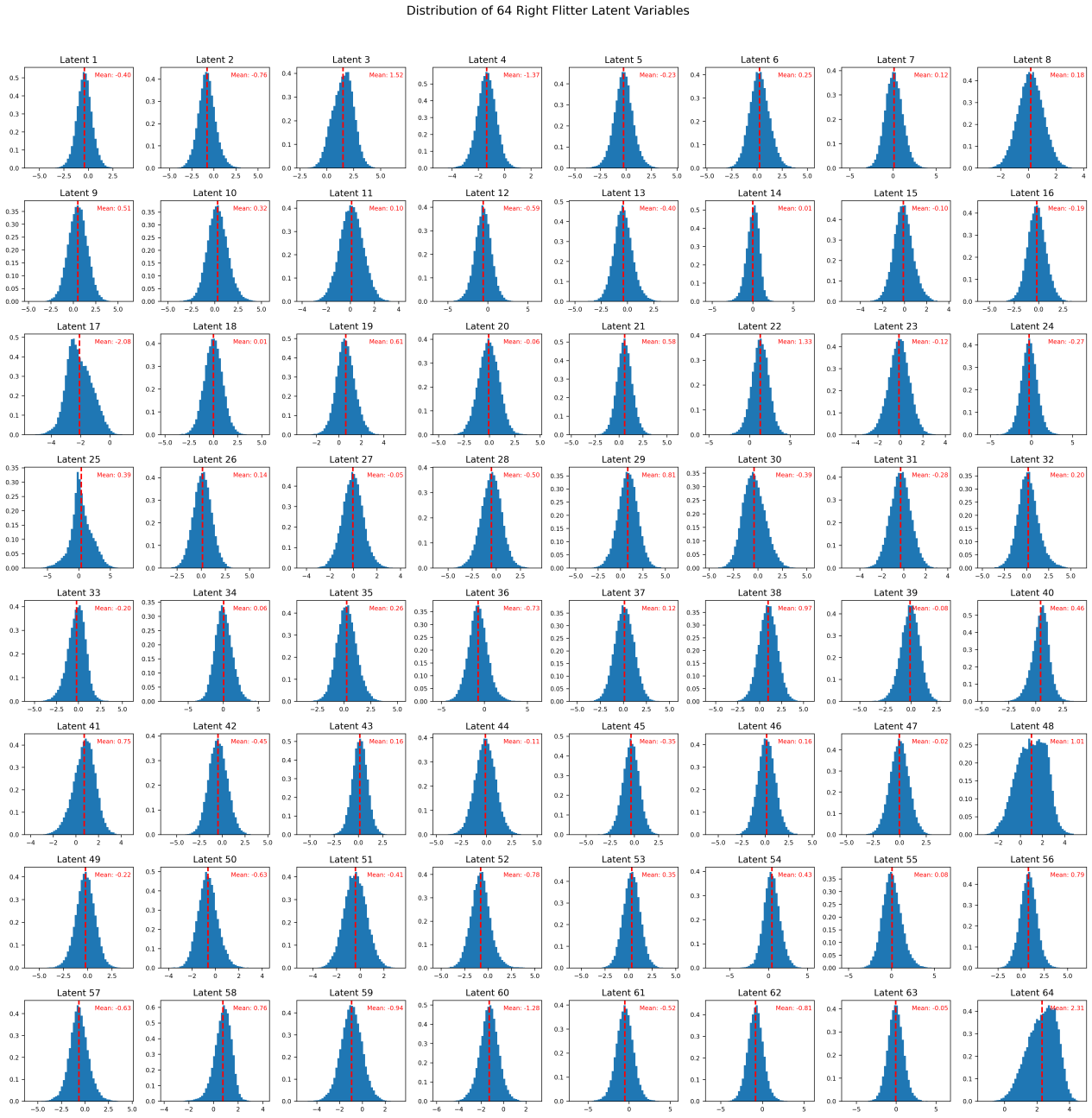


Fig C. The data distribution of right eye background IDFs


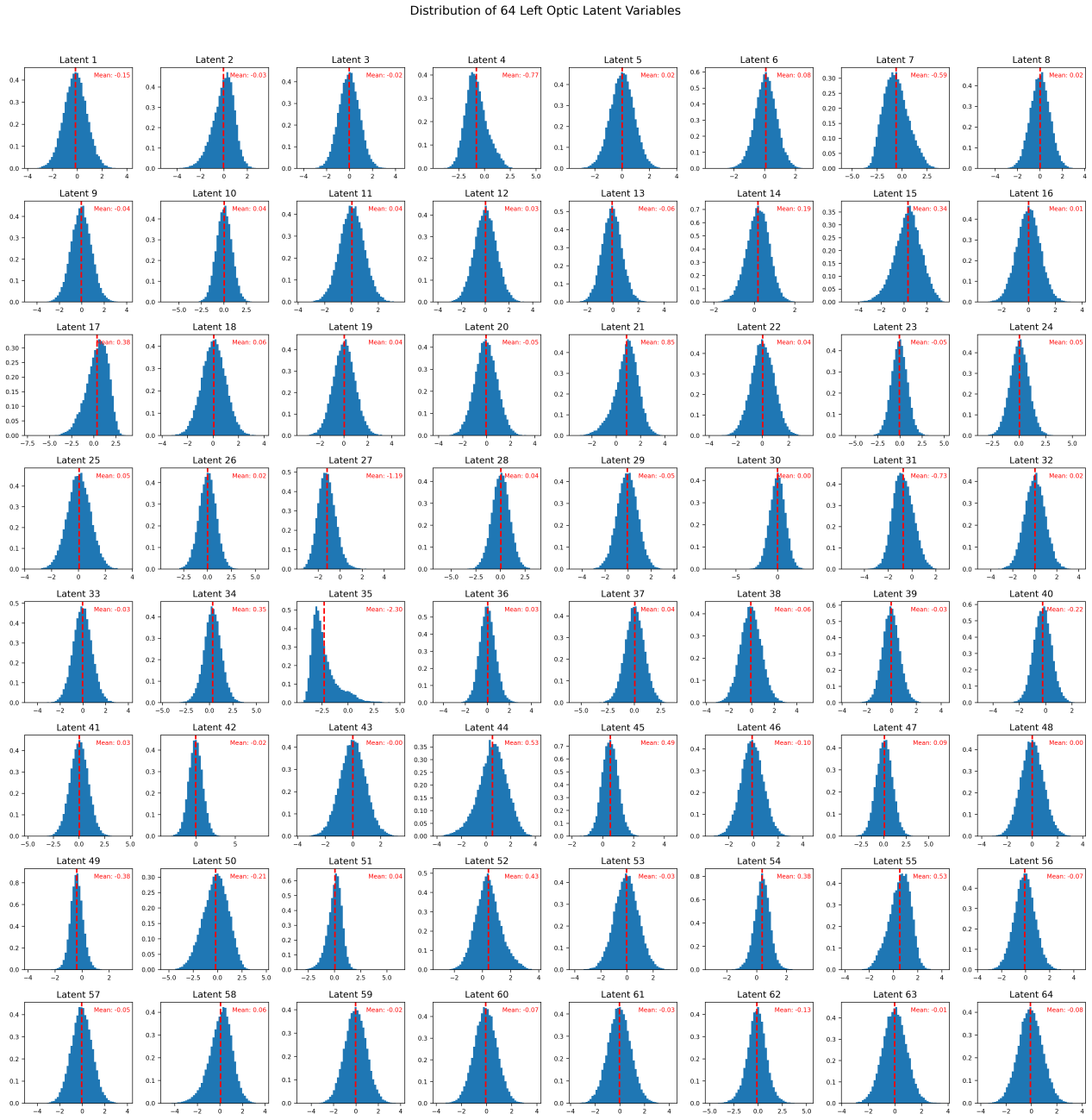


Fig D. The data distribution of left eye optic cup and disc IDFs


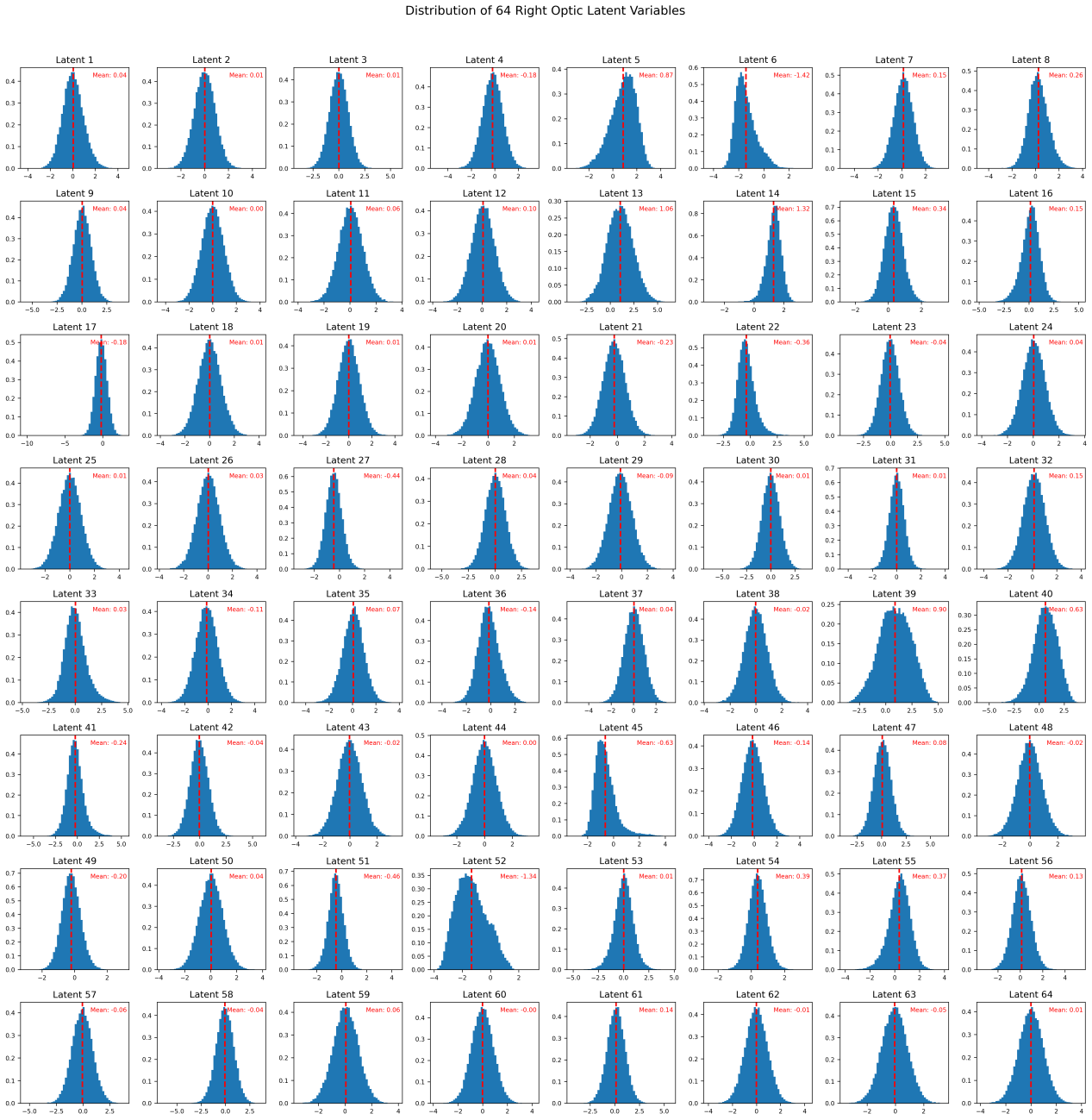


Fig E. The data distribution of right eye optic cup and disc IDFs


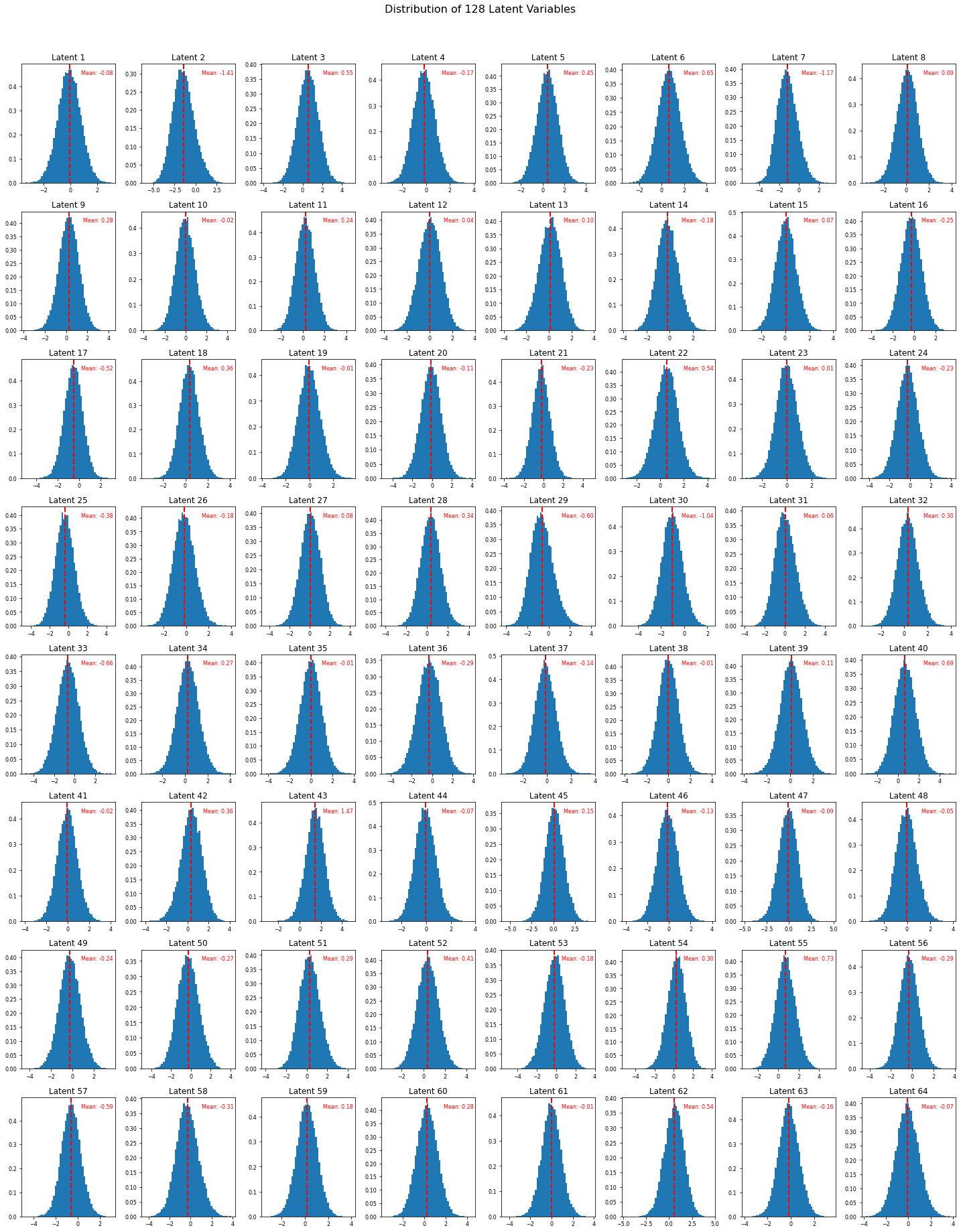
 Fig F. The data distribution of left eye vessel IDFs


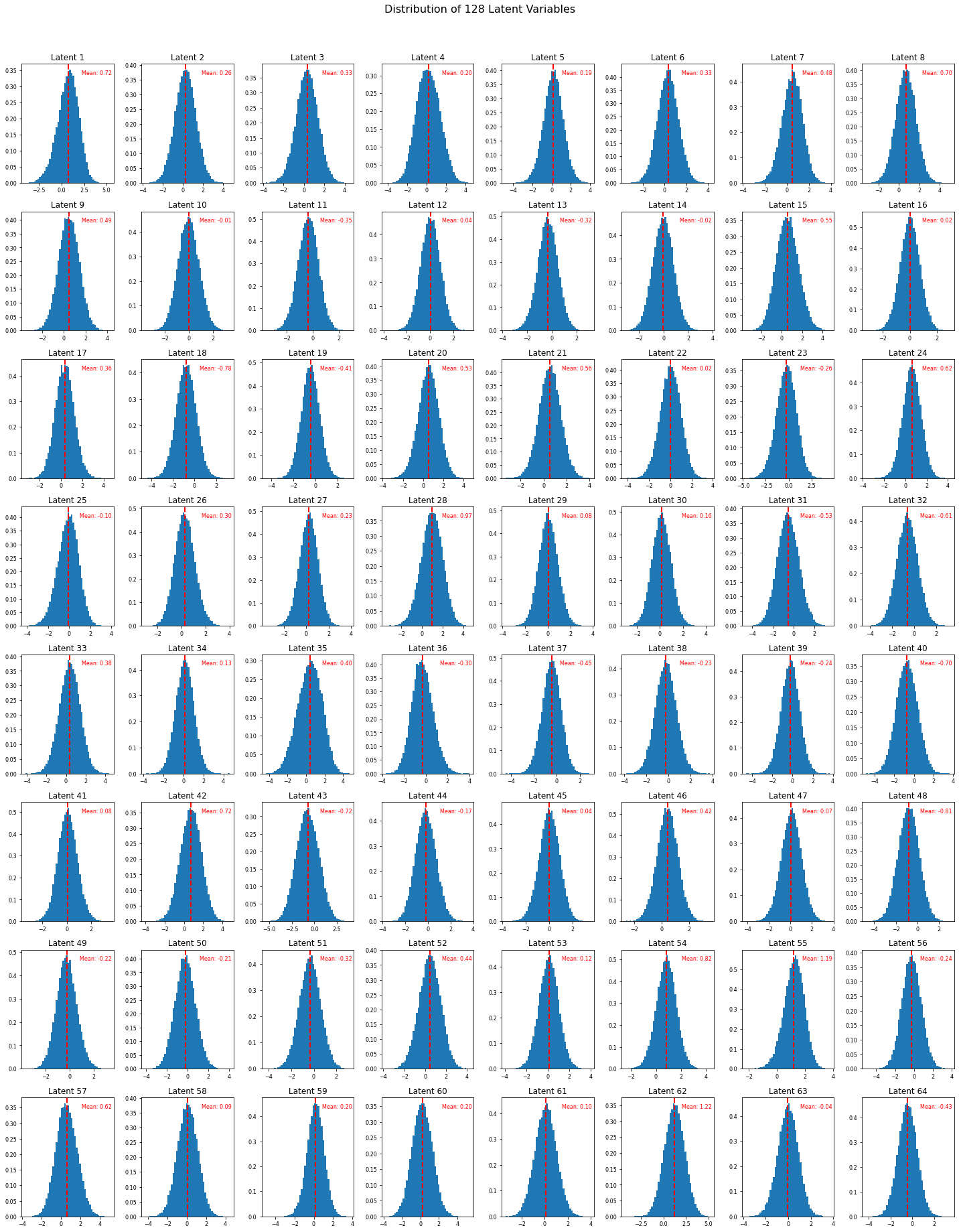
 Fig G. The data distribution of right eye vessel IDFs


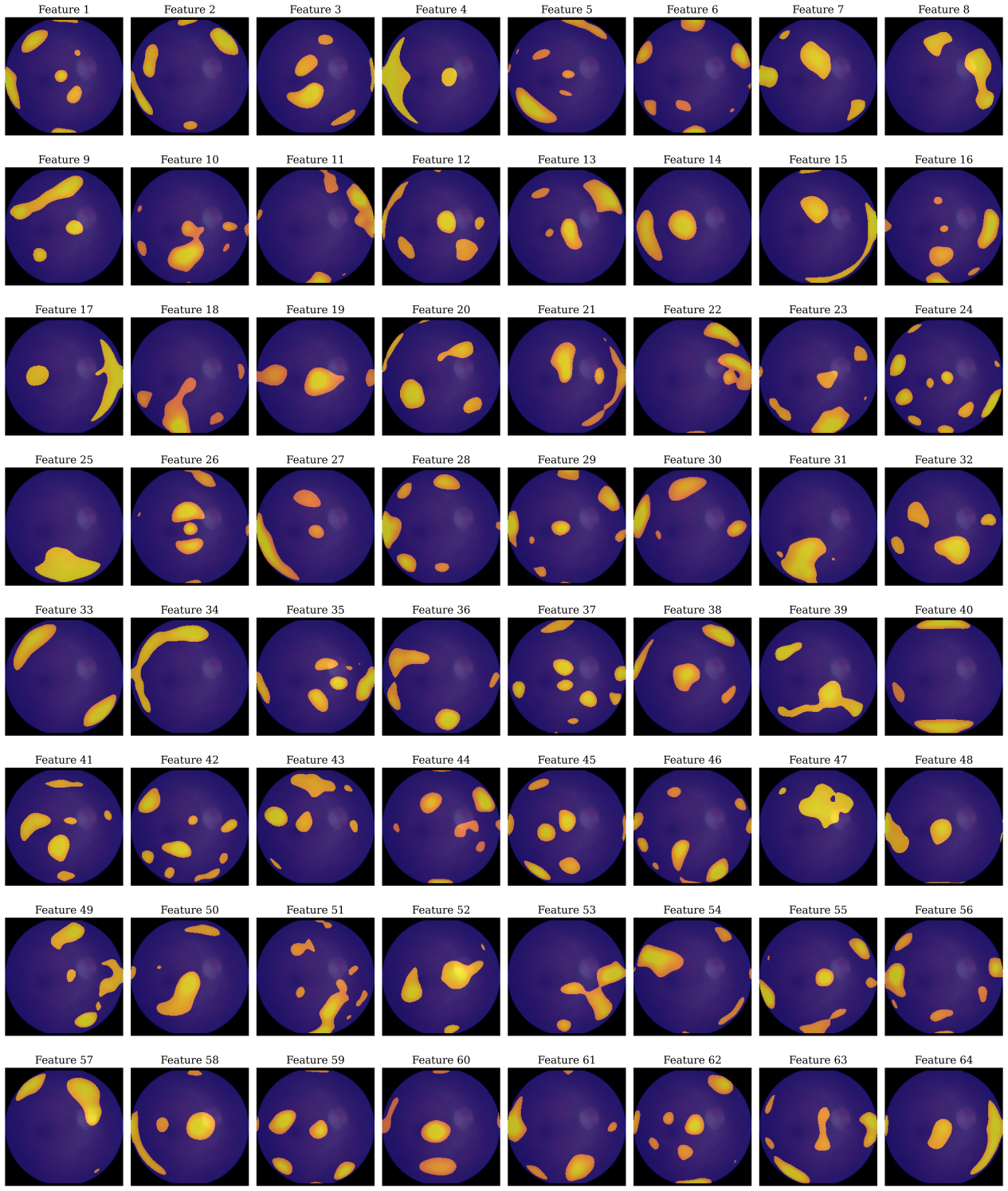


Fig H. Results of Fundus Background Perturbation Experiment


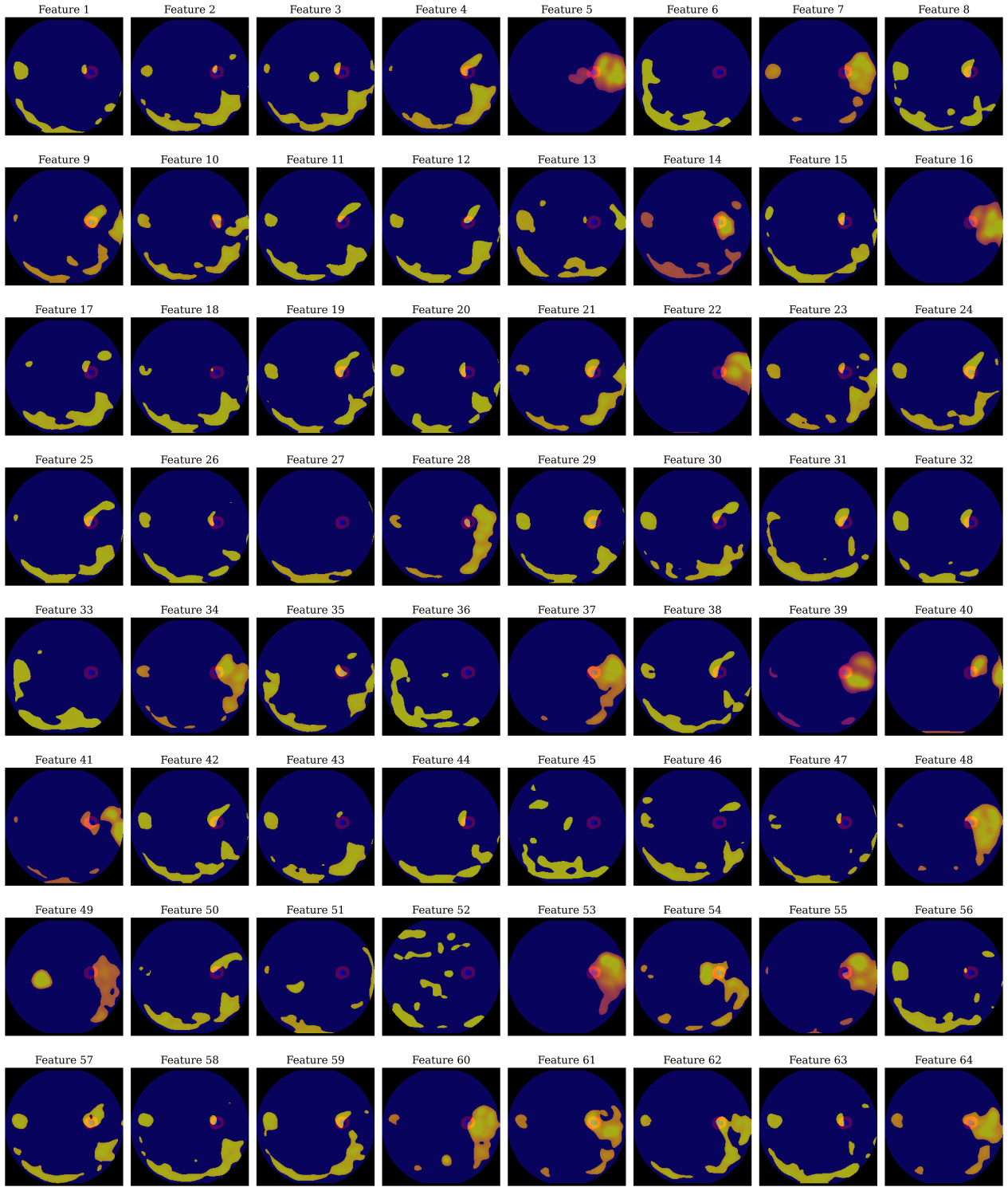
 Fig I. Results of Fundus Optic Cup and Disc Perturbation Experiment





Fig J. Results of Fundus Optic Cup and Disc Perturbation Experiment

**
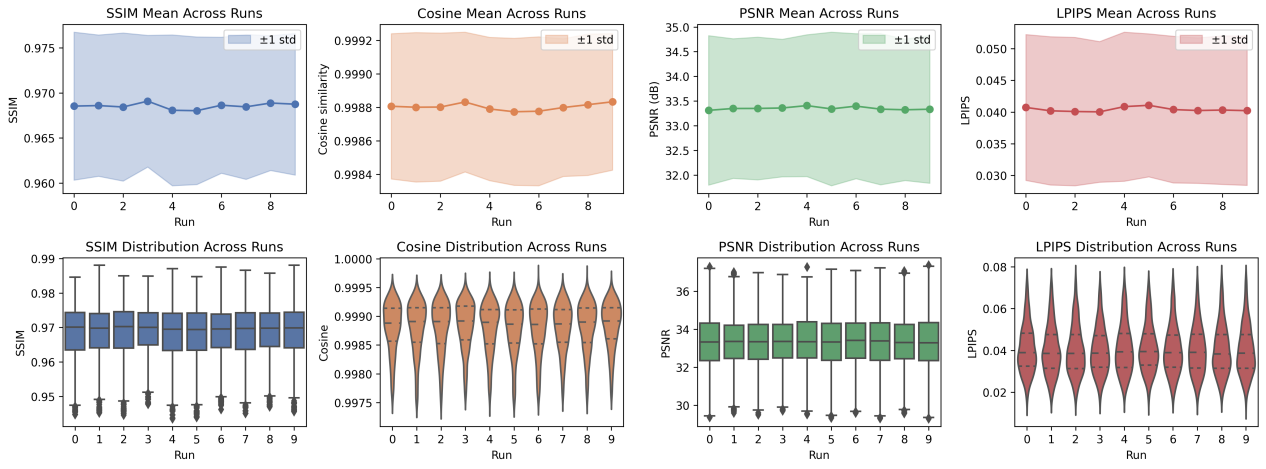
**

Fig K. Quantitative analysis of Left Fundus Background Features

**
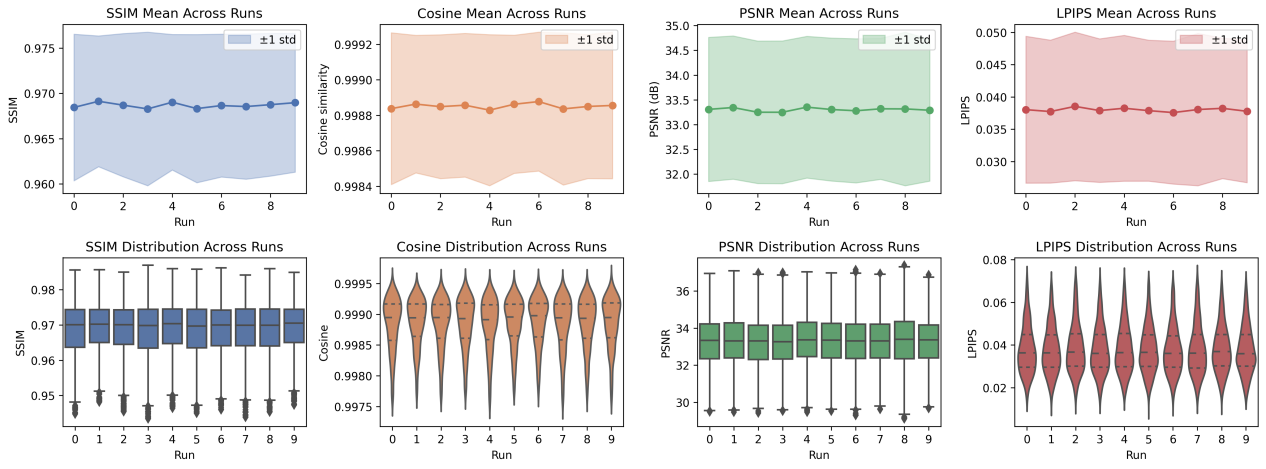
**

Fig L. Quantitative analysis of Left Fundus Background Features

**
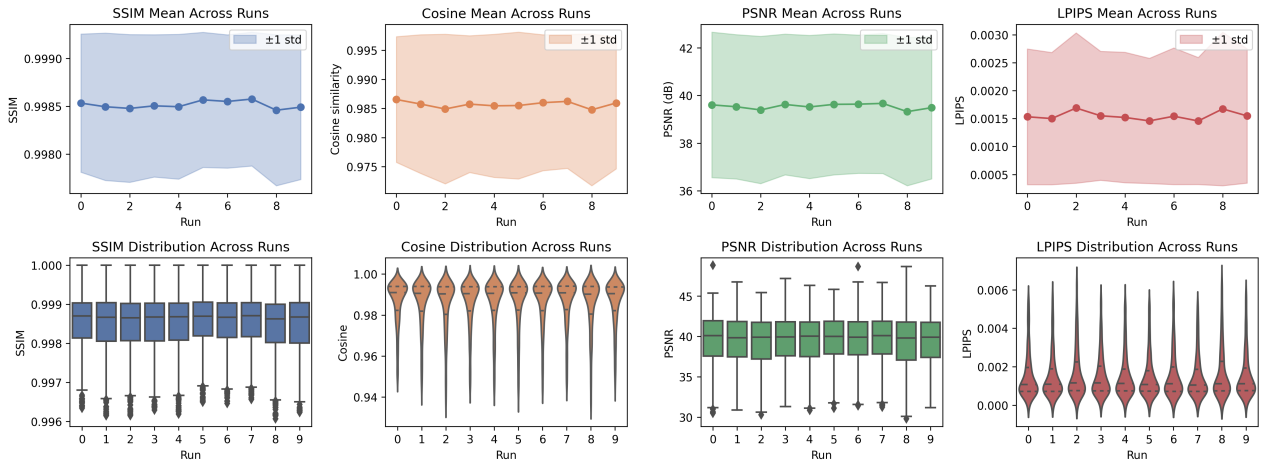
**

Fig M. Quantitative analysis of Left Fundus Optic Cup and Disc

**
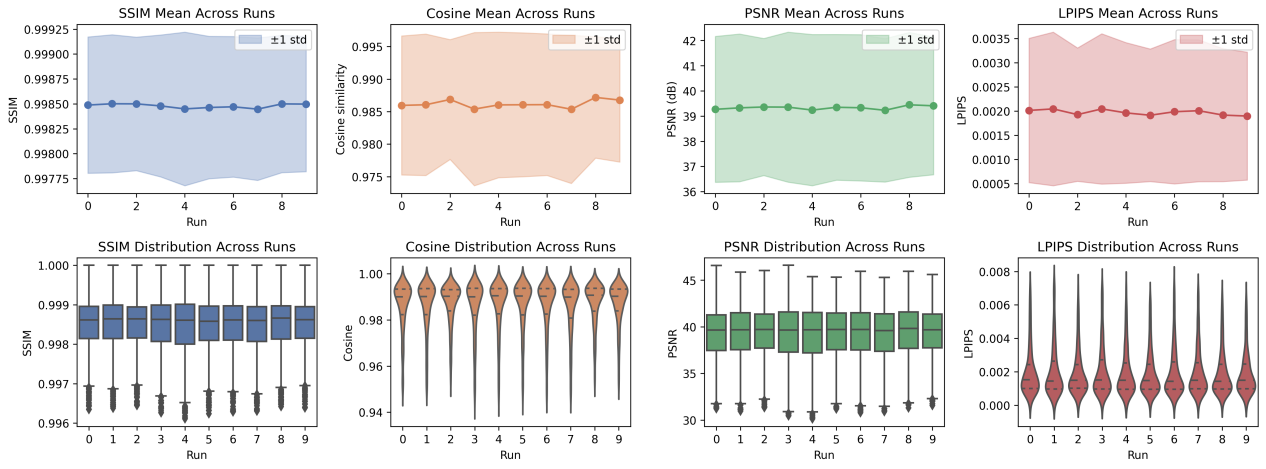
**

Fig N. Quantitative analysis of Right Fundus Optic Cup

**
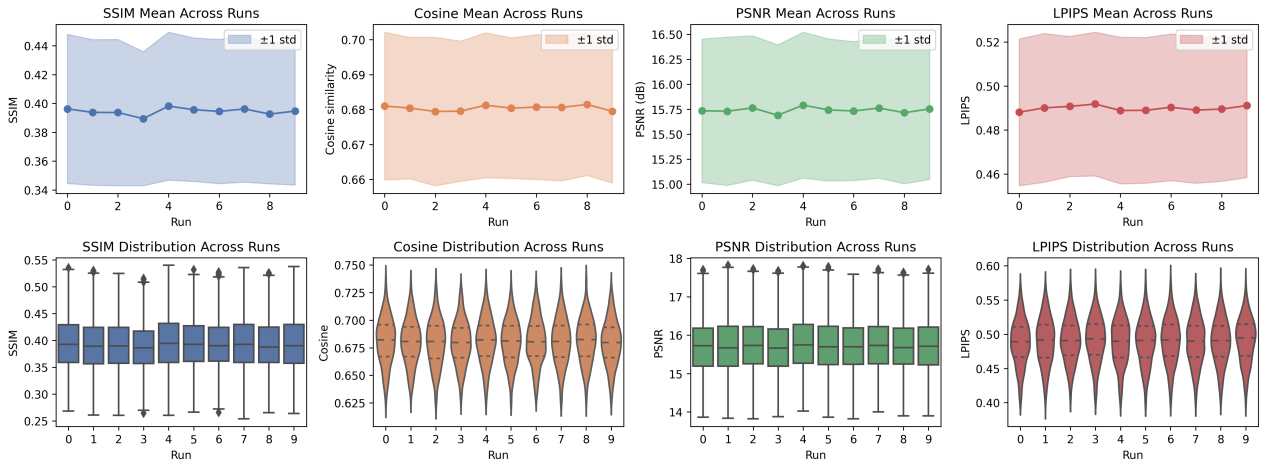
**

Fig O. Quantitative analysis of Left Fundus Vessel

**
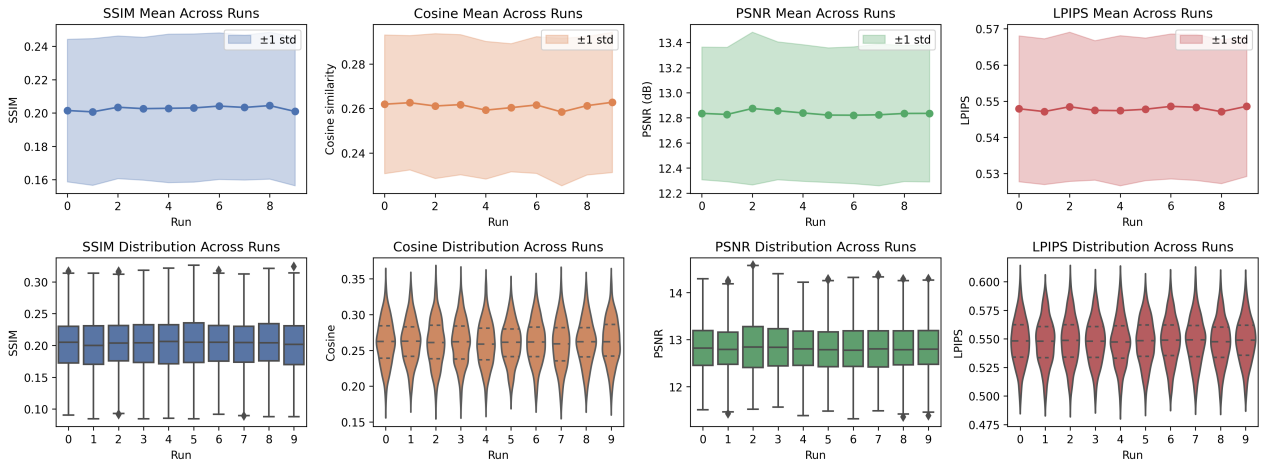
**

Fig P. Quantitative analysis of Right Fundus Vessel

**
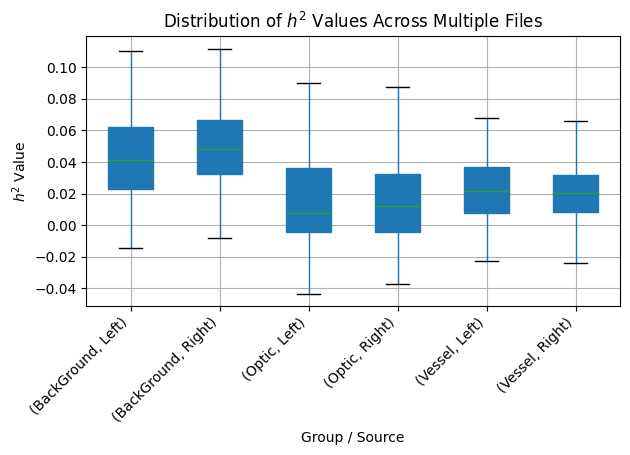
**

Fig Q. Distribution of h^2^ Values Across Multiple Latent Features

**
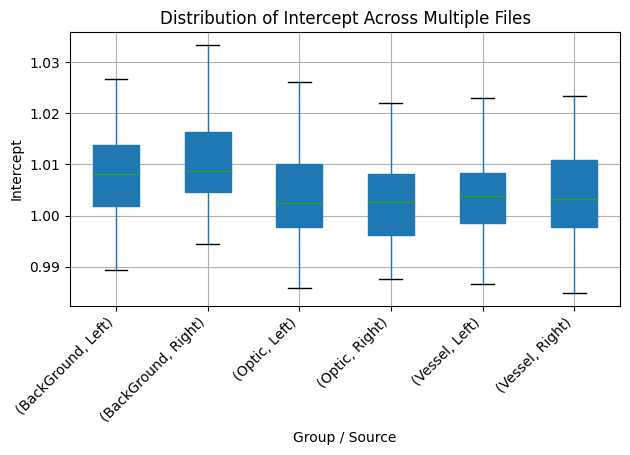
**

Fig R. Distribution of Intercept Across Multiple Latent Features

**
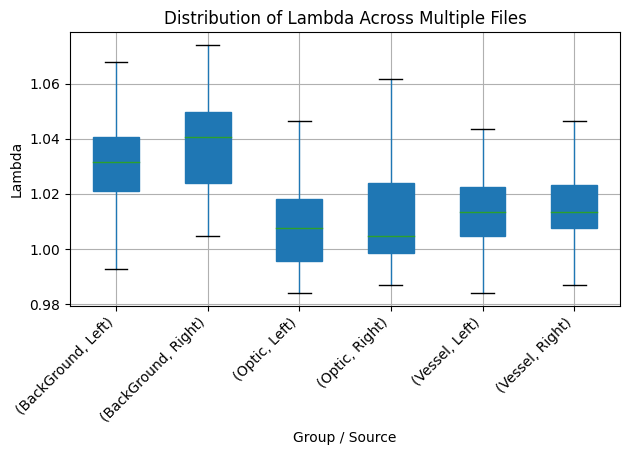
**

Fig S. Distribution of Lambda Across Multiple Latent Features


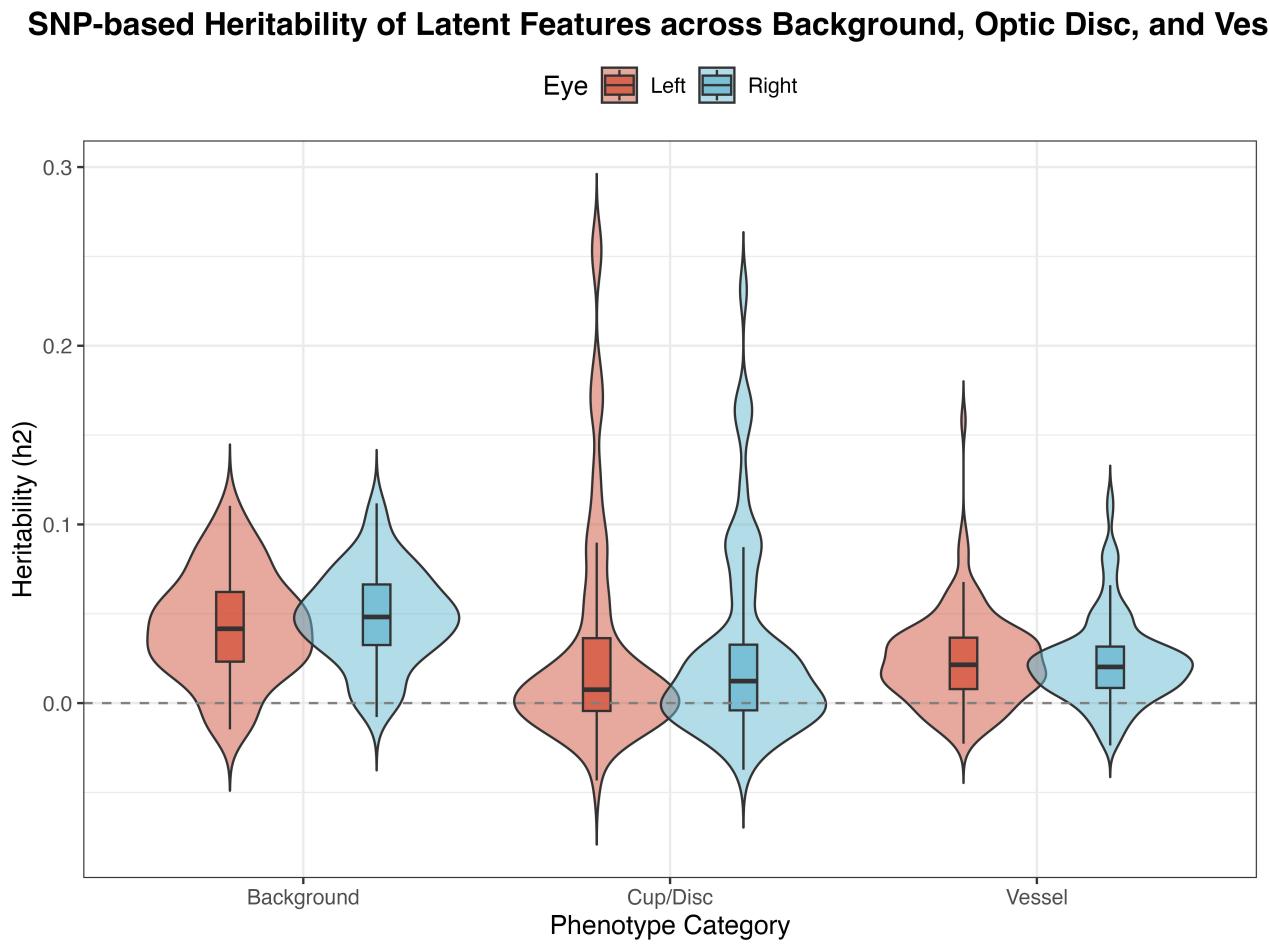


Fig T. SNP-based Heritability of Latent Features across Three Substructures


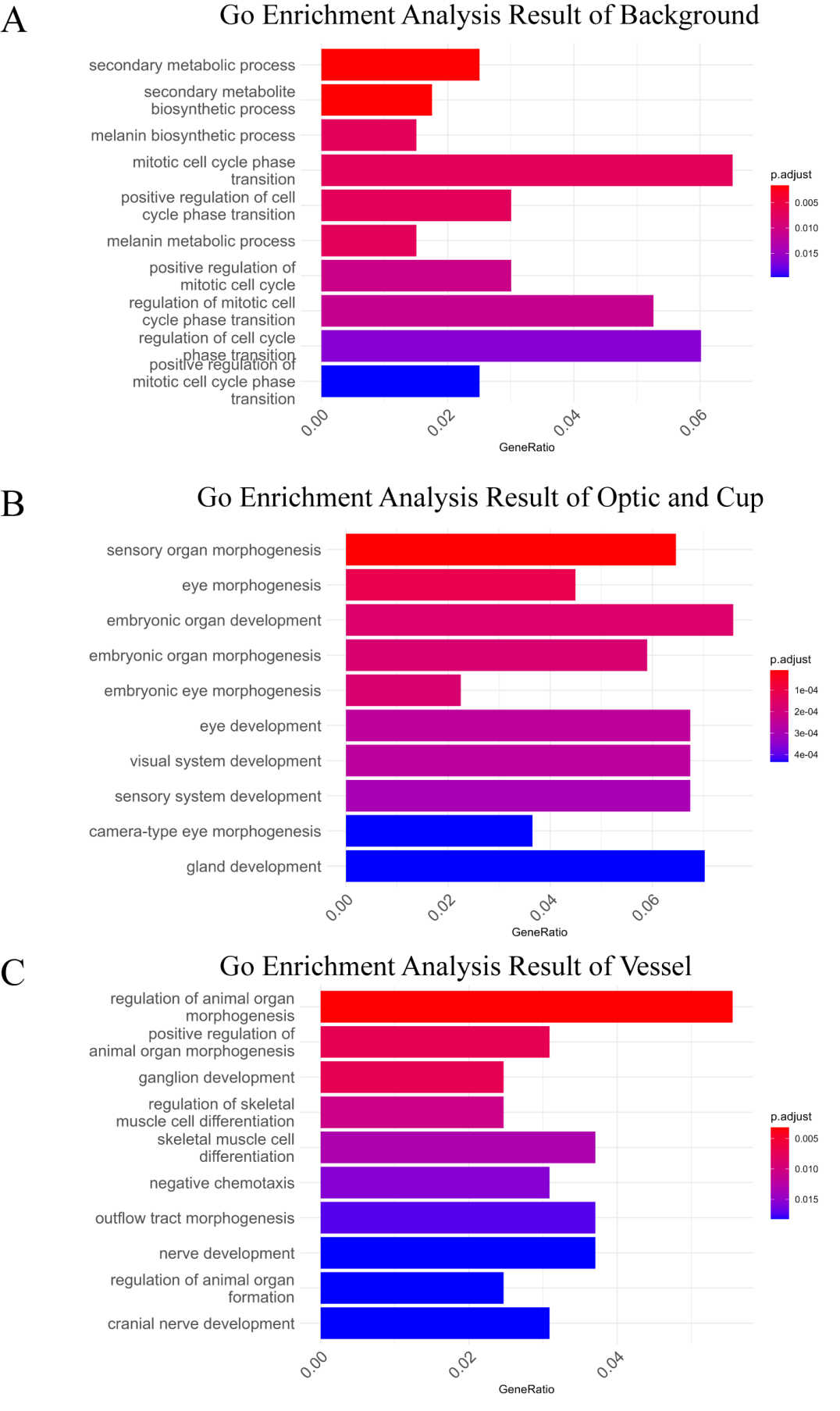


Fig U. GP Enrichment Analysis
