# Supplementary material for: Structure-aware retinal disentanglement reveals the genetic architecture of ocular and systemic diseases
Source: PLOS Digit Health. 2026 May 15;5(5):e0001376. doi: 10.1371/journal.pdig.0001376 (PMC13178874; doi:10.1371/journal.pdig.0001376)
Supplement: S3 File — Table A. 15 External Validation Dataset Information. Table B. Comparison of Reconstruction Fidelity Between the Proposed SA-VAE Framework and the Monolithic Baseline. Table C. Quantitative Evaluation of Disentangled Latent Representations. Table D–Table L. Summary of C-GWAS Loci. Table M. Significant Results of LDSC Analysis. (DOCX) [file pdig.0001376.s003.docx]

**Structure-Aware Retinal Disentanglement Reveals the Genetic Architecture of Ocular and Systemic Diseases**

**Tables**

**Contents**

Table A: 15 External Validation Dataset Information

Table B. Comparison of Reconstruction Fidelity Between the Proposed SA-VAE Framework and the Monolithic Baseline

Table C. Quantitative Evaluation of Disentangled Latent Representations

Table D–Table L. Summary of C-GWAS Loci

Table M. Significant Results of LDSC Analysis

**Table A. 15 External Validation Dataset Information**

| **Data** | **Disease** | **Number** | **URL** |
| --- | --- | --- | --- |
| IDRID | DR/NA | 517 | https://idrid.grand-challenge.org/ |
| messidor | DR | 1750 | https://www.adcis.net/en/third-party/messidor2/ |
| AMDM | AMD/NA | 1201 | https://aistudio.baidu.com/datasetdetail/177184 |
| FGADR | DR/NA | 1842 | https://drive.grand-challenge.org/ |
| MuReD | 39Diseases | 2208 | https://data.mendeley.com/datasets/pc4mb3h8hz/1 |
| PALM | PM/NA | 1200 | https://palm.grand-challenge.org/ |
| APTOS | DR/NA | 5590 | https://tianchi.aliyun.com/dataset/dataDetail?dataId=120007 |
| AOD | 8Diseases | 14813 | https://www.kaggle.com/datasets/nurmukhammed7/augemnted-ocular-diseases |
| JSIEC | 29Diseases | 997 | https://www.kaggle.com/datasets/linchundan/fundusimage1000 |
| Retina | 4Diseases | 601 | https://github.com/cvblab/retina_dataset |
| Toxoplasmosis | 5Diseases | 409 | https://www.kaggle.com/datasets/andrewmvd/ocular-toxoplasmosis-fundus-images-dataset |
| Diabetic_Retinopathy_Arranged_datasets | DR/NA | 35126 | https://www.kaggle.com/datasets/way2tutorials/diabetic-retinopathy-dataset |
| ODIR5k | 8Diseases | 6393 | https://odir2019.grand-challenge.org/ |
| RFMID | 45Diseases | 3185 | https://zenodo.org/records/7505822 |
| AIROGS | GL/NA | 101443 | https://airogs.grand-challenge.org/ |

**Table B. Comparison of Reconstruction Fidelity Between the Proposed SA-VAE Framework and the Monolithic Baseline**

**Table C. Quantitative Evaluation of Disentangled Latent Representations**


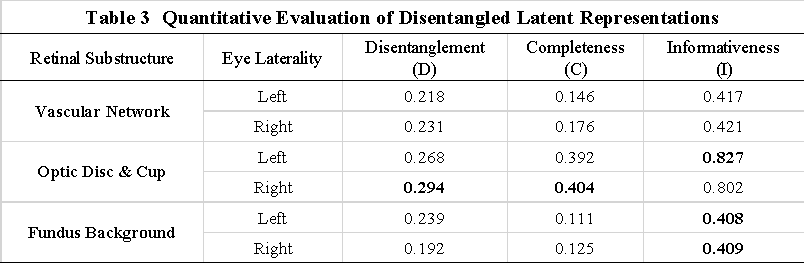


**Table D Summary of Left Eye Background C-GWAS Loci**

| **Locus ID** | **Chr** | **Lead SNP** | **P-value** | **Start** | **End** | **Number of SNPs** |
| --- | --- | --- | --- | --- | --- | --- |
| 1 | 1 | rs7512999 | 3.15E-10 | 87641352 | 87652415 | 19 |
| 2 | 1 | rs2493210 | 2.74E-10 | 92077097 | 92094044 | 5 |
| 3 | 1 | rs61820950 | 3.17E-08 | 202687486 | 202798831 | 22 |
| 4 | 1 | rs6687271 | 3.91E-24 | 205023028 | 205269501 | 348 |
| 5 | 2 | rs60843830 | 5.35E-42 | 30762 | 305803 | 257 |
| 6 | 3 | rs9844200 | 2.20E-08 | 14383370 | 14385513 | 4 |
| 7 | 3 | rs9837662 | 4.89E-08 | 181342947 | 181373642 | 23 |
| 8 | 4 | rs2101698 | 1.91E-10 | 81257721 | 81768583 | 315 |
| 9 | 5 | rs72759609 | 1.88E-08 | 31952051 | 31968777 | 9 |
| 10 | 6 | rs12174727 | 8.39E-15 | 403799 | 557932 | 95 |
| 11 | 6 | rs1207782 | 5.38E-09 | 22056923 | 22098047 | 37 |
| 12 | 6 | rs4897180 | 3.89E-16 | 126623947 | 127080700 | 274 |
| 13 | 6 | rs9376000 | 4.45E-16 | 134324910 | 134345361 | 30 |
| 14 | 6 | rs4869773 | 1.63E-08 | 153258023 | 153348742 | 10 |
| 15 | 7 | rs4719573 | 1.40E-08 | 19548842 | 19654201 | 240 |
| 16 | 8 | rs12547986 | 1.35E-12 | 61951918 | 62002751 | 87 |
| 17 | 9 | rs1325118 | 5.94E-18 | 12512207 | 12775488 | 416 |
| 18 | 9 | rs62550802 | 7.64E-10 | 76503064 | 76648938 | 11 |
| 19 | 10 | rs3858146 | 2.88E-41 | 69912278 | 70324814 | 838 |
| 20 | 11 | rs1455114 | 1.58E-09 | 15993057 | 16229382 | 147 |
| 21 | 11 | rs72928978 | 1.66E-45 | 68811777 | 69061635 | 225 |
| 22 | 11 | rs1486873 | 3.52E-15 | 87773405 | 87932015 | 107 |
| 23 | 11 | rs1126809 | 4.67E-93 | 88224550 | 90029884 | 1199 |
| 24 | 12 | rs5442 | 1.28E-12 | 6954864 | 6997808 | 2 |
| 25 | 12 | rs7953905 | 3.26E-15 | 20601010 | 20711134 | 30 |
| 26 | 12 | rs9971729 | 6.46E-09 | 23929026 | 23979791 | 18 |
| 27 | 12 | rs77783810 | 1.95E-17 | 96129363 | 96313248 | 139 |
| 28 | 13 | rs9561596 | 3.00E-22 | 95094385 | 95207513 | 204 |
| 29 | 14 | rs1959449 | 1.61E-10 | 54478412 | 54801587 | 166 |
| 30 | 14 | rs7493429 | 4.24E-32 | 60786981 | 61583340 | 263 |
| 31 | 14 | rs9989152 | 1.79E-08 | 68761015 | 68815513 | 49 |
| 32 | 14 | rs194755 | 5.92E-09 | 69212043 | 69227099 | 9 |
| 33 | 14 | rs887595 | 4.49E-17 | 74465825 | 74685975 | 257 |
| 34 | 15 | rs72714143 | 5.21E-240 | 27819158 | 28566862 | 502 |
| 35 | 15 | rs2671016 | 3.46E-16 | 29010369 | 29058544 | 40 |
| 36 | 15 | rs12912104 | 1.41E-13 | 29332198 | 29332198 | 1 |
| 37 | 15 | rs9210 | 2.43E-08 | 75033400 | 75230502 | 80 |
| 38 | 16 | rs7188859 | 4.88E-09 | 7459347 | 7462994 | 9 |
| 39 | 17 | rs8070929 | 1.61E-53 | 79429358 | 79690196 | 623 |
| 40 | 18 | rs4940460 | 1.01E-10 | 57045134 | 57115133 | 17 |
| 41 | 20 | rs16994801 | 2.71E-12 | 14513492 | 14689146 | 129 |
| 42 | 22 | rs1547014 | 1.85E-18 | 28540492 | 29133033 | 199 |
| 43 | 22 | rs563402269 | 2.55E-15 | 46362822 | 46383612 | 11 |

**Table E. Summary of Right Eye Background C-GWAS Loci**

| **Locus ID** | **Chr** | **Lead SNP** | **P-value** | **Start** | **End** | **Number of SNPs** |
| --- | --- | --- | --- | --- | --- | --- |
| 1 | 1 | rs12021948 | 1.78E-10 | 3042834 | 3056222 | 29 |
| 2 | 1 | rs4379694 | 1.43E-09 | 19820158 | 19973920 | 68 |
| 3 | 1 | rs7512999 | 1.20E-18 | 87641352 | 87652415 | 19 |
| 4 | 1 | rs1192419 | 1.77E-11 | 92041515 | 92094044 | 18 |
| 5 | 1 | rs72710406 | 1.73E-08 | 172471071 | 172604371 | 5 |
| 6 | 1 | rs3862948 | 6.19E-24 | 205022977 | 205269501 | 359 |
| 7 | 1 | rs340864 | 9.79E-09 | 214101186 | 214139159 | 31 |
| 8 | 2 | rs2290911 | 1.70E-29 | 30762 | 305803 | 257 |
| 9 | 2 | rs13015001 | 5.13E-15 | 145413855 | 145627526 | 158 |
| 10 | 2 | rs7567351 | 9.26E-12 | 208730387 | 208822924 | 21 |
| 11 | 2 | rs112067424 | 6.87E-09 | 239187209 | 239372632 | 112 |
| 12 | 3 | rs10510562 | 7.60E-09 | 25354513 | 25412484 | 56 |
| 13 | 3 | rs9842371 | 2.05E-10 | 181342947 | 181373642 | 23 |
| 14 | 4 | rs1807771 | 1.44E-09 | 81419735 | 81768583 | 62 |
| 15 | 6 | rs12174727 | 6.35E-21 | 403799 | 557932 | 143 |
| 16 | 6 | rs4145443 | 4.54E-14 | 21445615 | 23056810 | 66 |
| 17 | 6 | rs4374796 | 2.41E-09 | 73577780 | 73643289 | 28 |
| 18 | 6 | rs9398787 | 2.64E-11 | 126040435 | 126122280 | 64 |
| 19 | 6 | rs1591805 | 1.39E-20 | 126623947 | 127331951 | 555 |
| 20 | 6 | rs12193446 | 7.24E-13 | 129820038 | 129820038 | 1 |
| 21 | 6 | rs9376000 | 7.89E-17 | 134324910 | 134345361 | 30 |
| 22 | 6 | rs9478897 | 3.20E-10 | 151283211 | 151323664 | 90 |
| 23 | 7 | rs1962615 | 1.10E-09 | 28504010 | 28550394 | 23 |
| 24 | 7 | rs11769886 | 2.06E-10 | 99894971 | 100099466 | 191 |
| 25 | 8 | rs12547986 | 4.16E-18 | 61951918 | 62002751 | 87 |
| 26 | 8 | rs6473086 | 1.56E-15 | 78841080 | 79003651 | 185 |
| 27 | 8 | rs6469158 | 1.19E-08 | 88904358 | 89083083 | 21 |
| 28 | 8 | rs620272 | 5.34E-13 | 109081660 | 109346827 | 206 |
| 29 | 9 | rs62538954 | 3.99E-22 | 12536037 | 12775488 | 414 |
| 30 | 9 | rs4645630 | 4.53E-08 | 21996623 | 22077543 | 68 |
| 31 | 9 | rs11143748 | 1.06E-11 | 76454263 | 76710503 | 15 |
| 32 | 10 | rs1900003 | 2.33E-36 | 69912278 | 70312849 | 841 |
| 33 | 10 | rs10887264 | 1.93E-08 | 86009171 | 86019863 | 12 |
| 34 | 11 | rs1455118 | 7.19E-16 | 15993057 | 16229382 | 147 |
| 35 | 11 | rs6484500 | 1.94E-15 | 31132257 | 31813529 | 493 |
| 36 | 11 | rs72928978 | 3.85E-44 | 68811777 | 69061635 | 261 |
| 37 | 11 | rs61901598 | 1.58E-14 | 87861777 | 87932015 | 92 |
| 38 | 11 | rs1126809 | 1.56E-88 | 88224550 | 89533159 | 2045 |
| 39 | 12 | rs5442 | 3.84E-22 | 6954864 | 6997808 | 2 |
| 40 | 12 | rs7295612 | 5.62E-14 | 20601010 | 20711134 | 38 |
| 41 | 12 | rs10771034 | 4.29E-09 | 23929026 | 23979791 | 18 |
| 42 | 12 | rs80247972 | 5.86E-20 | 96129363 | 96313248 | 197 |
| 43 | 13 | rs876133 | 1.27E-10 | 34002488 | 34094345 | 141 |
| **Table F. Summary of Right Eye Background C-GWAS Loc** | | | | | | |
| **Locus ID** | **Chr** | **Lead SNP** | **P-value** | **Start** | **End** | **Number of SNPs** |
| 44 | 13 | rs9534252 | 2.35E-08 | 46499784 | 46510333 | 5 |
| 45 | 13 | rs2197568 | 2.56E-08 | 69766455 | 69773910 | 39 |
| 46 | 13 | rs7334485 | 4.70E-28 | 95094385 | 95207513 | 204 |
| 47 | 13 | rs4772261 | 3.64E-08 | 100645723 | 100724485 | 15 |
| 48 | 14 | rs1957622 | 2.15E-11 | 54444750 | 54801587 | 321 |
| 49 | 14 | rs10146342 | 2.39E-32 | 60786981 | 61583340 | 271 |
| 50 | 14 | rs7148979 | 4.63E-21 | 74465825 | 74687417 | 312 |
| 51 | 15 | rs72714143 | 2.98E-262 | 27888348 | 28566862 | 647 |
| 52 | 15 | rs2671016 | 1.22E-09 | 29010369 | 29058544 | 40 |
| 53 | 15 | rs12912104 | 5.25E-12 | 29332198 | 29332198 | 1 |
| 54 | 15 | rs589135 | 6.69E-12 | 34989626 | 35009676 | 41 |
| 55 | 15 | rs72746905 | 6.66E-09 | 84315251 | 84486669 | 74 |
| 56 | 15 | rs6496560 | 2.54E-08 | 89723370 | 89774325 | 69 |
| 57 | 16 | rs369930774 | 2.65E-16 | 7456936 | 7462994 | 14 |
| 58 | 17 | rs2908972 | 8.14E-09 | 11395418 | 11437291 | 13 |
| 59 | 17 | rs9912396 | 3.79E-21 | 68673837 | 68735692 | 95 |
| 60 | 17 | rs8070929 | 1.86E-65 | 79429358 | 79690196 | 738 |
| 61 | 19 | rs8103067 | 2.62E-09 | 32023850 | 32038701 | 23 |
| 62 | 21 | rs8131841 | 3.84E-09 | 44776815 | 44799734 | 37 |
| 63 | 21 | rs2839019 | 9.75E-11 | 47041808 | 47412511 | 249 |
| 64 | 22 | rs5762753 | 3.48E-24 | 28552395 | 29446611 | 183 |
| 65 | 22 | rs9330813 | 1.46E-13 | 46362822 | 46383612 | 11 |

**Table G. Summary of Left Eye Optic Cup and Disc C-GWAS Loci**

| **Locus ID** | **Chr** | **Lead SNP** | **P-value** | **Start** | **End** | **Number of SNPs** |
| --- | --- | --- | --- | --- | --- | --- |
| 1 | 1 | rs199755205 | 2.47E-08 | 68863119 | 68908501 | 78 |
| 2 | 1 | rs1192415 | 6.44E-37 | 92025038 | 92094044 | 132 |
| 3 | 1 | rs12137699 | 1.83E-10 | 116208598 | 116251926 | 19 |
| 4 | 1 | rs116366530 | 7.06E-10 | 227126969 | 227677723 | 259 |
| 5 | 2 | rs11883726 | 6.20E-11 | 56166398 | 56392173 | 107 |
| 6 | 2 | rs1529387 | 1.93E-08 | 145413855 | 145597193 | 112 |
| 7 | 2 | rs72892835 | 2.30E-10 | 172527238 | 172933248 | 106 |
| 8 | 2 | rs11691125 | 3.69E-08 | 208785303 | 208822924 | 18 |
| 9 | 3 | rs6787363 | 4.47E-10 | 25368413 | 25459108 | 57 |
| 10 | 3 | rs9843102 | 1.64E-11 | 100601059 | 100709716 | 44 |
| 11 | 4 | rs906566 | 2.35E-08 | 112299090 | 112516065 | 100 |
| 12 | 5 | rs72759609 | 7.01E-09 | 31952051 | 31968777 | 9 |
| 13 | 6 | rs10499118 | 2.57E-09 | 122388851 | 122712377 | 218 |
| 14 | 6 | rs4989144 | 1.15E-08 | 166590870 | 166615078 | 12 |
| 15 | 8 | rs12547986 | 1.23E-20 | 61951918 | 62002751 | 87 |
| 16 | 8 | rs36142508 | 1.81E-08 | 109060667 | 109244742 | 240 |
| 17 | 9 | rs4645630 | 6.13E-15 | 21995882 | 22125347 | 151 |
| 18 | 10 | rs3858145 | 1.84E-91 | 69900418 | 70595426 | 1248 |
| 19 | 11 | rs74918256 | 1.37E-08 | 2145768 | 2172830 | 19 |
| 20 | 11 | rs35131353 | 4.27E-08 | 29135383 | 29139477 | 3 |
| 21 | 11 | rs72928978 | 6.72E-23 | 68811777 | 68964137 | 165 |
| 22 | 11 | rs570817202 | 4.68E-14 | 88536451 | 89058101 | 414 |
| 23 | 12 | rs11048785 | 1.02E-08 | 27096233 | 27404656 | 79 |
| 24 | 12 | rs11115958 | 2.90E-18 | 83865479 | 84226327 | 561 |
| 25 | 12 | rs80247972 | 9.19E-20 | 96129363 | 96313248 | 139 |
| 26 | 12 | rs838327 | 3.03E-08 | 114920632 | 114939288 | 14 |
| 27 | 13 | rs9509847 | 7.43E-09 | 22294062 | 22323519 | 25 |
| 28 | 13 | rs142132426 | 1.02E-08 | 42678357 | 42809961 | 49 |
| 29 | 13 | rs9561619 | 3.95E-09 | 95153167 | 95207513 | 185 |
| 30 | 14 | rs1177257 | 2.12E-08 | 35920211 | 35969438 | 8 |
| 31 | 14 | rs1254319 | 1.70E-18 | 60786981 | 61583340 | 254 |
| 32 | 14 | rs7142674 | 6.27E-13 | 74465825 | 74666641 | 255 |
| 33 | 15 | rs61756152 | 8.14E-48 | 28043390 | 28566862 | 181 |
| 34 | 15 | rs62012628 | 1.17E-08 | 79030526 | 79082431 | 37 |
| 35 | 16 | rs4332760 | 2.04E-09 | 7459347 | 7462994 | 9 |
| 36 | 16 | rs8053277 | 1.19E-12 | 51455794 | 51659244 | 53 |
| 37 | 17 | rs9912699 | 1.05E-11 | 58898689 | 59289905 | 275 |
| 38 | 18 | rs11080461 | 4.14E-08 | 1079268 | 1098517 | 11 |
| 39 | 18 | rs1893789 | 2.65E-12 | 56988930 | 57128486 | 67 |
| 40 | 22 | rs1972202 | 5.13E-23 | 28540492 | 29133033 | 191 |
| 41 | 22 | rs5756825 | 2.05E-11 | 37908435 | 38220132 | 118 |
| 42 | 22 | rs75792643 | 2.44E-13 | 46362822 | 46383612 | 11 |

**Table H. Summary of Right Eye Optic Cup and Disc C-GWAS Loci**

| **Locus ID** | **Chr** | **Lead SNP** | **P-value** | **Start** | **End** | **Number of SNPs** |
| --- | --- | --- | --- | --- | --- | --- |
| 1 | 1 | rs1192415 | 92077097 | 7.90E-52 | 91085207 | 92766912 |
| 2 | 1 | rs12143283 | 110631977 | 2.34E-08 | 110525630 | 110637394 |
| 3 | 1 | rs12136690 | 116208944 | 2.97E-08 | 116208598 | 116251926 |
| 4 | 1 | rs11811115 | 205134090 | 3.25E-10 | 205024573 | 205216110 |
| 5 | 1 | rs116657151 | 227530925 | 1.07E-19 | 227156620 | 227677723 |
| 6 | 2 | rs1561208 | 5689697 | 1.84E-08 | 5689487 | 5692633 |
| 7 | 2 | rs13399945 | 56239897 | 1.38E-09 | 56204789 | 56257941 |
| 8 | 2 | rs1561369 | 183702964 | 1.10E-08 | 183700883 | 183704681 |
| 9 | 2 | rs2718666 | 208796905 | 1.76E-08 | 208785303 | 208822924 |
| 10 | 2 | rs11682290 | 218605498 | 1.71E-08 | 218511592 | 218616633 |
| 11 | 3 | rs12629250 | 25048410 | 1.31E-08 | 25034135 | 25051556 |
| 12 | 3 | rs35667547 | 64547477 | 4.21E-11 | 64547477 | 64547477 |
| 13 | 3 | rs9833999 | 99147683 | 3.25E-08 | 99047293 | 99152366 |
| 14 | 3 | rs142287284 | 100627655 | 1.35E-16 | 100601059 | 100799713 |
| 15 | 3 | rs4681374 | 147476878 | 6.10E-09 | 147435898 | 147491975 |
| 16 | 5 | rs72759609 | 31952051 | 9.00E-13 | 31952051 | 31968777 |
| 17 | 5 | rs16903234 | 87833801 | 9.26E-10 | 87782483 | 87919700 |
| 18 | 6 | rs9392026 | 453415 | 9.18E-09 | 453415 | 453415 |
| 19 | 6 | rs1361107 | 126767511 | 7.72E-12 | 126657472 | 127080700 |
| 20 | 6 | rs1324130 | 140391106 | 7.07E-13 | 140217063 | 140836911 |
| 21 | 6 | rs116853146 | 151561936 | 7.08E-09 | 151552375 | 151575944 |
| 22 | 7 | rs536201576 | 72066140 | 8.62E-10 | 71982261 | 72308840 |
| 23 | 8 | rs12547986 | 61986668 | 7.69E-21 | 61951918 | 62002751 |
| 24 | 8 | rs10081494 | 88725316 | 1.06E-10 | 88602399 | 88784385 |
| 25 | 8 | rs77706046 | 122709203 | 4.32E-08 | 122697620 | 122733968 |
| 26 | 9 | rs7865618 | 22031005 | 5.59E-25 | 21974218 | 22125503 |
| 27 | 9 | rs11143745 | 76600134 | 4.48E-09 | 76503064 | 76648938 |
| 28 | 10 | rs61854803 | 70014638 | 3.87E-81 | 69900776 | 70595426 |
| 29 | 10 | rs12771867 | 104246691 | 6.59E-12 | 104222963 | 104487443 |
| 30 | 11 | rs58590292 | 31744269 | 6.89E-11 | 31132257 | 31791099 |
| 31 | 11 | rs72928978 | 68831364 | 1.42E-14 | 68811777 | 69034247 |
| 32 | 11 | rs4081455 | 88891543 | 4.06E-22 | 88358544 | 89058101 |
| 33 | 12 | rs7952844 | 83988636 | 8.14E-31 | 83858153 | 84228697 |
| 34 | 12 | rs80247972 | 96270298 | 8.63E-29 | 96089737 | 96364340 |
| 35 | 13 | rs506409 | 22318352 | 4.72E-09 | 22294062 | 22323519 |
| 36 | 13 | rs1570621 | 47170118 | 2.41E-09 | 47067841 | 47192049 |
| 37 | 13 | rs9556398 | 95193822 | 6.10E-09 | 95153167 | 95207513 |
| 38 | 13 | rs837324 | 101175879 | 2.64E-10 | 100803855 | 101259427 |
| 39 | 14 | rs1033732 | 60829564 | 2.22E-18 | 60786981 | 61160190 |
| 40 | 14 | rs11159069 | 74657063 | 2.78E-15 | 74465825 | 74666641 |
| 41 | 15 | rs61756152 | 28412872 | 4.00E-53 | 28044716 | 28566862 |
| 42 | 15 | rs7167736 | 101206638 | 9.95E-15 | 101200873 | 101210973 |
| 43 | 16 | rs7188859 | 7460426 | 1.09E-13 | 7456936 | 7462994 |
| **Table I. Summary of Right Eye Optic Cup and Disc C-GWAS Loci** | | | | | | |
| **Locus ID** | **Chr** | **Lead SNP** | **P-value** | **Start** | **End** | **Number of SNPs** |
| 44 | 16 | rs1420993 | 51464898 | 9.03E-14 | 51393548 | 51659244 |
| 45 | 17 | rs9893705 | 59269555 | 5.52E-13 | 58898689 | 59309754 |
| 46 | 17 | rs9912396 | 68726544 | 6.28E-09 | 68695559 | 68735692 |
| 47 | 17 | rs8070929 | 79530993 | 8.56E-13 | 79526569 | 79686552 |
| 48 | 18 | rs8091667 | 395394 | 2.40E-08 | 395394 | 395394 |
| 49 | 18 | rs1975499 | 57100410 | 8.57E-09 | 57066107 | 57128995 |
| 50 | 19 | rs8103067 | 32027415 | 2.61E-09 | 32023850 | 32038701 |
| 51 | 22 | rs1547014 | 29100711 | 1.85E-31 | 28540492 | 29446975 |
| 52 | 22 | rs1534913 | 37918672 | 1.48E-14 | 37906262 | 38399979 |
| 53 | 22 | rs73175083 | 46383612 | 6.23E-16 | 46362822 | 46387180 |

**Table J. Summary of Left Eye Vessel C-GWAS Loci**

| **Locus ID** | **Chr** | **Lead SNP** | **P-value** | **Start** | **End** | **Number of SNPs** |
| --- | --- | --- | --- | --- | --- | --- |
| 1 | 1 | rs34085461 | 4.01E-12 | 182579035 | 182581157 | 4 |
| 2 | 2 | rs2177489 | 5.86E-09 | 48778115 | 49158655 | 83 |
| 3 | 2 | rs1529386 | 3.36E-08 | 145413855 | 145601057 | 117 |
| 4 | 2 | rs264658 | 4.86E-12 | 159808696 | 159983207 | 115 |
| 5 | 2 | rs111676152 | 5.00E-11 | 172527238 | 172933248 | 103 |
| 6 | 3 | rs76001234 | 1.42E-12 | 77174165 | 77271362 | 79 |
| 7 | 4 | rs6845966 | 4.33E-08 | 96214829 | 96299093 | 45 |
| 8 | 4 | rs16999566 | 1.53E-08 | 166978941 | 167003769 | 43 |
| 9 | 5 | rs115803211 | 2.22E-24 | 87100442 | 88065637 | 694 |
| 10 | 5 | rs13159421 | 1.64E-13 | 179402769 | 179579682 | 108 |
| 11 | 6 | rs13194747 | 2.12E-08 | 140249466 | 140736134 | 297 |
| 12 | 7 | rs4732544 | 1.65E-14 | 83640062 | 83799292 | 203 |
| 13 | 8 | rs10958686 | 4.08E-08 | 41237437 | 41375246 | 66 |
| 14 | 8 | rs7816434 | 1.82E-27 | 109013996 | 109447132 | 679 |
| 15 | 9 | rs62550802 | 5.78E-12 | 76503064 | 76648938 | 11 |
| 16 | 9 | rs12378141 | 1.46E-08 | 77017768 | 77159265 | 44 |
| 17 | 9 | rs34627198 | 3.64E-08 | 90895634 | 91901342 | 256 |
| 18 | 10 | rs118180052 | 1.48E-08 | 11958946 | 12090909 | 187 |
| 19 | 10 | rs10762201 | 9.99E-19 | 69934467 | 70266659 | 526 |
| 20 | 10 | rs7902564 | 2.52E-08 | 102626032 | 102633551 | 22 |
| 21 | 11 | rs79744168 | 1.61E-08 | 58174653 | 58417882 | 189 |
| 22 | 12 | rs11045245 | 3.71E-09 | 20577805 | 20589390 | 10 |
| 23 | 12 | rs77369040 | 1.98E-34 | 96129363 | 96380753 | 360 |
| 24 | 12 | rs11059501 | 4.55E-08 | 128566031 | 128572894 | 11 |
| 25 | 13 | rs9554663 | 2.47E-08 | 100648356 | 100787374 | 45 |
| 26 | 14 | rs59813981 | 2.47E-09 | 24861049 | 24865526 | 4 |
| 27 | 14 | rs10873114 | 2.85E-16 | 59636749 | 59806027 | 111 |
| 28 | 14 | rs1254328 | 1.00E-36 | 60786981 | 61583340 | 554 |
| 29 | 14 | rs887595 | 3.47E-17 | 74465825 | 74669893 | 305 |
| 30 | 15 | rs28579204 | 1.33E-08 | 71733820 | 71739969 | 3 |
| 31 | 16 | rs72792792 | 1.24E-09 | 78644807 | 78694695 | 46 |
| 32 | 17 | rs9909684 | 8.18E-11 | 13480862 | 13510421 | 18 |
| 33 | 17 | rs11077605 | 2.39E-08 | 70375639 | 70390452 | 25 |
| 34 | 18 | rs3810040 | 2.91E-12 | 352087 | 463418 | 46 |
| 35 | 18 | rs62080075 | 3.40E-08 | 38165433 | 38334884 | 28 |
| 36 | 18 | rs1893789 | 3.35E-11 | 57062358 | 57128486 | 51 |
| 37 | 18 | rs6566272 | 1.46E-08 | 64889611 | 65012844 | 171 |
| 38 | 20 | rs200846794 | 1.08E-20 | 14513492 | 14811477 | 173 |
| 39 | 22 | rs73175083 | 1.03E-10 | 46362822 | 46387180 | 12 |

**Table K. Summary of Right Eye Vessel C-GWAS Loci**

| **Locus ID** | **Chr** | **Lead SNP** | **P-value** | **Start** | **End** | **Number of SNPs** |
| --- | --- | --- | --- | --- | --- | --- |
| 1 | 1 | rs993099 | 4.49E-17 | 82355255 | 82559524 | 109 |
| 2 | 1 | rs16837415 | 1.93E-08 | 156443232 | 156443232 | 1 |
| 3 | 1 | rs34085461 | 2.12E-13 | 182579035 | 182581157 | 4 |
| 4 | 2 | rs11684168 | 2.73E-08 | 88676478 | 88694167 | 3 |
| 5 | 2 | rs13015001 | 8.38E-10 | 145413855 | 145601439 | 118 |
| 6 | 2 | rs10173859 | 2.27E-08 | 159951710 | 159954696 | 6 |
| 7 | 2 | rs4666845 | 3.91E-08 | 183353325 | 183414262 | 35 |
| 8 | 2 | rs13030484 | 4.56E-10 | 218396460 | 218615511 | 187 |
| 9 | 3 | rs17006079 | 1.54E-15 | 69545919 | 69592250 | 63 |
| 10 | 3 | rs77652315 | 8.33E-13 | 77174165 | 77299434 | 155 |
| 11 | 3 | rs79050611 | 2.72E-08 | 78740009 | 79024541 | 45 |
| 12 | 3 | rs76629150 | 5.05E-09 | 107079716 | 107159469 | 21 |
| 13 | 4 | rs6536530 | 1.29E-08 | 161556711 | 161580174 | 10 |
| 14 | 4 | rs16999566 | 4.10E-08 | 166978941 | 167003769 | 43 |
| 15 | 5 | rs17421627 | 6.75E-25 | 87513775 | 87919700 | 320 |
| 16 | 5 | rs6864861 | 4.08E-08 | 126205155 | 126543309 | 275 |
| 17 | 5 | rs13160214 | 8.51E-10 | 179424244 | 179579682 | 83 |
| 18 | 6 | rs4559102 | 9.90E-12 | 126657472 | 127080700 | 250 |
| 19 | 6 | rs12193446 | 2.37E-10 | 129820038 | 129820038 | 1 |
| 20 | 6 | rs13194747 | 3.56E-10 | 140249466 | 140736134 | 297 |
| 21 | 7 | rs2372035 | 2.14E-10 | 83710376 | 83799292 | 173 |
| 22 | 7 | rs7787947 | 9.14E-09 | 84159768 | 84544961 | 317 |
| 23 | 8 | rs12547986 | 1.51E-11 | 61969213 | 62002590 | 46 |
| 24 | 8 | rs1553016 | 1.07E-08 | 85991064 | 86260295 | 83 |
| 25 | 8 | rs395815 | 2.26E-29 | 109012769 | 109584744 | 670 |
| 26 | 9 | rs10781177 | 1.79E-11 | 76503064 | 76648938 | 11 |
| 27 | 10 | rs11819716 | 2.80E-11 | 45143950 | 45247418 | 143 |
| 28 | 10 | rs10762198 | 9.00E-34 | 69934258 | 70266659 | 646 |
| 29 | 10 | rs78732656 | 3.11E-08 | 70605872 | 70778813 | 77 |
| 30 | 10 | rs2817690 | 2.50E-09 | 98857923 | 98898413 | 57 |
| 31 | 11 | rs2996467 | 1.50E-09 | 31434854 | 31792295 | 33 |
| 32 | 12 | rs78633929 | 3.08E-32 | 96095781 | 96380753 | 402 |
| 33 | 12 | rs10850065 | 1.21E-09 | 109772975 | 109848567 | 140 |
| 34 | 13 | rs9585316 | 3.17E-12 | 100647612 | 101259427 | 309 |
| 35 | 14 | rs17095853 | 3.01E-12 | 59626385 | 59806027 | 109 |
| 36 | 14 | rs1010053 | 1.68E-40 | 60769259 | 61583340 | 288 |
| 37 | 14 | rs7142674 | 8.64E-23 | 74269694 | 74685975 | 468 |
| 38 | 15 | rs524952 | 3.62E-08 | 34989626 | 35009875 | 36 |
| 39 | 15 | rs8036545 | 3.16E-10 | 101200873 | 101210973 | 11 |
| 40 | 16 | rs56292889 | 2.81E-09 | 6514588 | 6567812 | 45 |
| 41 | 16 | rs7191400 | 2.03E-10 | 23865986 | 23957370 | 23 |
| 42 | 16 | rs8055396 | 2.66E-10 | 51455794 | 51482321 | 14 |
| 43 | 16 | rs55863307 | 8.58E-09 | 80503223 | 80549413 | 45 |
| **Table L: Summary of Right Eye Vessel C-GWAS Loci** | | | | | | |
| Locus ID | Chr | Lead SNP | P-value | Start | End | Number of SNPs |
| 44 | 17 | rs9909684 | 1.50E-08 | 13480862 | 13510421 | 18 |
| 45 | 17 | rs12150267 | 5.21E-12 | 58813056 | 59289905 | 540 |
| 46 | 17 | rs717419 | 4.96E-08 | 68695559 | 68735692 | 38 |
| 47 | 17 | rs7208640 | 2.19E-08 | 76568716 | 76573179 | 10 |
| 48 | 18 | rs2621198 | 4.95E-08 | 458662 | 493780 | 7 |
| 49 | 18 | rs8092290 | 2.09E-10 | 56945392 | 57128832 | 132 |
| 50 | 19 | rs1644659 | 5.15E-09 | 37376756 | 37815381 | 397 |
| 51 | 20 | rs11700265 | 4.29E-15 | 14513492 | 14689146 | 145 |
| 52 | 22 | rs77164166 | 2.59E-11 | 46362822 | 46383612 | 11 |
